# Synthetic ACTH in High Risk Patients with Idiopathic Membranous Nephropathy: A Prospective, Open Label Cohort Study

**DOI:** 10.1371/journal.pone.0142033

**Published:** 2015-11-12

**Authors:** Anne-Els van de Logt, Charles H. Beerenhout, Hans S. Brink, Jos J. van de Kerkhof, Jack F. Wetzels, Julia M. Hofstra

**Affiliations:** 1 Radboud university medical center, Radboud Institute for Health Sciences, Department of Nephrology, Nijmegen, The Netherlands; 2 Maxima medical center, Department of Internal Medicine, Veldhoven, The Netherlands; 3 Medisch Spectrum Twente, Department of Internal Medicine, Enschede, The Netherlands; 4 Bernhoven Hospital, Department of Internal Medicine, Uden, The Netherlands; Ichan School of Medicine at Mount Sinai, UNITED STATES

## Abstract

New therapeutic agents are warranted in idiopathic membranous nephropathy. Synthetic ACTH may be advantageous with reported remission rates up to 85% and few side effects. We conducted a prospective open label cohort study from 2008 till 2010 (NCT00694863). We prospectively selected patients with idiopathic membranous nephropathy and high risk for progression (defined as βeta-2-microglobulin (β2m) excretion of >500 ng/min). For comparison, we selected matched historical controls treated with cyclophosphamide. The prospectively selected patients received intramuscular injections of synthetic ACTH during 9 months (maximal dose 1 mg twice a week). The primary endpoints concerned the feasibility and incidence of remissions as a primary event. Secondary endpoints included side effects of treatment and the incidence of remissions and relapses at long-term follow-up. Twenty patients (15 men) were included (age 54±14 years, serum creatinine 104 μmol/l [IQR 90–113], urine protein:creatinine ratio 8.7 g/10 mmol creatinine [IQR 4.3–11.1]). Seventeen patients (85%) completed treatment. 97% of injections were administered correctly. Cumulative remission rate was 55% (complete remission in 4 patients, partial remission 7 patients). In a group of historical controls treated with cyclophosphamide and steroids, 19 of 20 patients (95%) developed a remission (complete remission in 13 patients, partial remission in 6 patients) (p<0.01). The main limitation of our study is its small size and the use of a historical control group. We show that treatment with intramuscular injections of synthetic ACTH is feasible. Our data suggest that synthetic ACTH is less effective than cyclophosphamide in inducing a remission in high risk patients with idiopathic membranous nephropathy. The use of synthetic ACTH was also associated with many adverse events. Therefore, we advise against synthetic ACTH as standard treatment in membranous nephropathy.

**Trial Registration:**

ClinicalTrials.gov NCT00694863

## Introduction

Idiopathic membranous nephropathy (iMN) is a common cause of adult nephrotic syndrome. The recent discovery of anti-PLA2R antibodies in the majority of patients points to the autoimmune etiology of the disease. [1] Overall, approximately a third to up to half of patients develop a spontaneous remission whereas the other part develops renal failure, often after a prolonged period of stable but persistent proteinuria. [2–4]

Therapy in idiopathic membranous nephropathy remains debated. Two randomized controlled trials evaluated the efficacy of treatment with alkylating agents in patients with iMN of recent onset, with normal renal function and nephrotic range proteinuria. [5, 6] Immunosuppressive therapy increased remission rate and improved renal survival. However, in up to 50% of untreated patients outcome was favorable. This means that up to 50% of patients were unnecessarily exposed to treatment with toxic alkylating agents. Ideally, immunosuppressive treatment should be restricted to patients with iMN at high risk for ESRD.

The most recent KDIGO guidelines recommend restricting initial therapy with 6 months of cyclic steroids and cyclophosphamide (CP) to high-risk patients, defined as patients with persisting nephrotic syndrome (>6 months), deteriorating kidney function during the first 6–12 months after diagnosis, or severe symptoms related to nephrotic syndrome. [7] Still, the serious side effects of alkylating agents remain a concern. [8] Therefore, new non-toxic therapeutic agents are warranted.

In 1999 Berg et al. described beneficial effects of synthetic ACTH in 14 patients with nephrotic syndrome due to iMN. [9] Synthetic ACTH was administered to lower lipids in patients with hyperlipoproteinemia, which is one of the features of nephrotic syndrome. Treatment with synthetic ACTH led to the expected changes in serum lipid profile, but in addition there was an unexpected 90% decrease of urinary albumin excretion. The proteinuria-lowering effects of synthetic ACTH were confirmed in further studies of the same group, with prolonged remission after a 2–11 months course of synthetic ACTH treatment in patients with several proteinuric diseases (iMN, Minimal Change Disease, Mesangioproliferative glomerulonephritis, Diabetic Nephropathy, Focal Segmental Sclerosis). [10] In a subsequent randomized controlled trial 32 nephrotic patients with iMN were treated with either synthetic ACTH for 12 months or with the standard 6 months cyclical regimen with an alkylating agent (chlorambucil or cyclophosphamide) and steroids. [11] Remission rates were similar in both groups (87 and 75% for ACTH and alkylating agents respectively) and treatment with ACTH only caused mild side effects. Of note, patients in this RCT were not selected to have a high risk profile. The promising results of the above-mentioned trials led to a renewed interest in ACTH as treatment for iMN. [12–14]

Because the experience with synthetic ACTH in patients with iMN and high risk for renal failure is limited, we conducted a prospective, open cohort study (NCT00694863) to assess the safety and efficacy of synthetic ACTH in those patients.

## Methods

A prospective, open label cohort study was conducted in our academic center from February 2008 till December 2010, with follow-up extended till June 2014 (S1, S2 and S3 checklists). This study was registered at clinicaltrials.gov (NCT00694863). The study protocol met criteria required by the Declaration of Helsinki and was approved by both the regional ethical board (CMO regio Arnhem-Nijmegen) and the national authority (CCMO), all patients gave written informed consent (S1 and S2 protocols). Our study was approved by the ethical committee (CMO Arnhem-Nijmegen) on May 22^nd^ 2008, and registered in the clinical trials registry (clinicaltrials.gov) at June 9^th^ 2008. Registering the trial in a register before it was approved by the ethical board was not yet required and no common use in those days. The first participant started with the study treatment on September 22^nd^ 2008. Of note, the standardized urine measurement of the first participant had taken place in February 2008 as part of routine patient care. After approval of the study in May 2008, the patient was considered eligible for the study based on the retrospective data from this measurement. Screening date therefore is considered to be February 2008 for this patient. All other patients were screened and enrolled between June 25^th^ 2008 and December 29^th^ 2010. There are no ongoing or related trials to this study. Patients were recruited in our university hospital or one of the referring regional hospitals. Participating patients were seen at our outpatient clinic for a standardized evaluation, as described before. [15] During that evaluation eligibility criteria for participation in the ACTH study were verified.

The study included adult patients (age 18–80 years) with biopsy proven MN. Eligible patients had to have nephrotic syndrome (proteinuria ≥ 3.5 g/day and serum albumin ≤ 30 g/l) despite conservative treatment, an eGFR > 60 ml/min/1.73m^2^, a high risk for ESRD, and a (relative) contraindication to our standard treatment with cyclophosphamide and steroids. High risk for ESRD was defined as an urinary βeta-2-microglobulin (β2m) excretion of >500 ng/min. [15] Relative contraindications to standard care were: young age (risk of infertility), older age (> 60 years; more side effects of cyclophosphamide could be expected), previous treatment with cyclophosphamide (to avoid high cumulative doses), and intolerance for cyclophosphamide or high dose steroids. In case they had a (relative) contraindication for standard treatment, later on, also patients with an eGFR > 40 ml/min/1.73m^2^ were included. We excluded patients with secondary MN. [16] Other exclusion criteria were pregnancy, lactation or inadequate use of contraceptives; evidence of underlying systemic diseases, active infection, or active peptic ulcer disease; clinical evidence of renal vein thrombosis; severe asthma, known hypersensitivities, and/or previous allergic reaction to synthetic ACTH.

Patients were treated with synthetic ACTH (tetracosatide hexaacetate, Synacthen Depot^®^, Novartis, Switzerland) 1mg/ml suspension for intramuscular injection. ACTH was administered in an increasing dose, starting with 1 mg every week and with a maximum of 1 mg twice a week after 8 weeks (detailed treatment scheme adapted from the group of Berg, M. Arnadottir personal communication, S1 Table). Maximum dose was continued for 18 weeks and tapered afterwards, with a total treatment period of nine months and 59 injections. Since anaphylactic reactions have been described with administration of ACTH and because this drug is not registered for routine use in our country, the ethical board required that all injections would be administered in our hospital and patients had to be observed for 30 minutes after injection.

All patients were aggressively treated to decrease blood pressure (target value 130/80 mmHg), primarily by using angiotensin-converting enzyme inhibitors and/or angiotensin receptor blockers. Statins were administered in case of hyperlipidemia. Anticoagulant drugs were started when serum albumin levels dropped below 20 g/l (local current practice). All patients were advised to use a moderately salt-restricted diet.

Follow-up time started from the moment of start of treatment. Since all injections were administered in our hospital by one of the researchers, patients were frequently seen during treatment. Adverse events were recorded at every visit. Clinical data and laboratory data were collected at months 1, 2, 3, 5, 7 and 9 and every three months afterwards, with a final study visit at 24 months. Patients were followed thereafter. Anti-PLA2R antibody measurement was not available at the moment of inclusion in our study, which started in 2008. Subjects were withdrawn from the study when renal function significantly deteriorated during treatment, defined as an increase in serum creatinine of >25% over baseline value, or an increase to an absolute value >135 μmol/l. In these cases standard immunosuppressive treatment, consisting of cyclophosphamide and steroids, was initiated.

The primary outcome parameters were the feasibility of intramuscular ACTH therapy administered in the hospital twice a week and the incidence of remissions as a primary event (either complete or partial). Feasibility was defined as the percentage of injections that was administered correctly (at the indicated moment ± one day) during the complete treatment period. Secondary endpoints included the number of patients that completed the study period and the patient self-reported impact/burden on daily life of the treatment (on a scale of 1 to 10 (10 = highest level of satisfaction), not validated). Furthermore the change in proteinuria and change in eGFR were assessed, as were side effects of treatment. At long-term follow-up the incidence of remissions, relapses and use of additional treatment were assessed.

To evaluate the efficacy of ACTH, we compared the results obtained in the ACTH group with data of matched historical controls. These historical controls were treated between 1998 and 2007 with cyclophosphamide (CP) (standard protocol 1.5 mg/kg/day for 12 months) and steroids [17], and were matched for serum creatinine, proteinuria, age, gender and previous immunosuppressive treatment.

### Definitions and calculations

To correct for inappropriate 24-hour urine collections, amount of proteinuria was expressed as protein-creatinine ratio (grams per 10 mmol of creatinine).

Complete remission (CR) was defined as a protein-creatinine ratio ≤0.2 g/10 mmol creatinine with stable kidney function, and partial remission (PR) was defined as protein-creatinine ratio <3.0 g/10 mmol creatinine with a reduction of >50% from baseline and stable kidney function. Achieving remission includes both partial and complete remission. Nephrotic range proteinuria was defined as a protein-creatinine ratio ≥3.0 g/10 mmol creatinine. Relapse was defined as nephrotic proteinuria and an increase of > 50% compared with the lowest value during remission. We defined progression as 1) a rise in serum creatinine >50%, 2) a rise in serum creatinine >25% and an absolute level ≥135 μmol/l or 3) the need for immunosuppressive therapy as judged by the treating physician. [18] An event was defined only after repeated confirmatory measurements. We estimated GFR by applying the 4-variable MDRD equation. [19]

### Statistical analyses

Data are presented as number (percent), means (± SD) or medians (interquartile ranges [IQR]) when appropriate. T-test, Mann-Whitney U test and chi-squared test were used for comparisons between and within groups. Data were analysed on an intention to treat approach. The cumulative probability of a clinical event was estimated according to Kaplan-Meier analysis and evaluated using a log-rank test. All statistics were performed using IBM SPSS software, version 20 (Chicago, IL). Differences were considered significant with p-value <0.05.

#### Patient inclusion

After inclusion of six patients an interim analysis was performed to evaluate the feasibility of the study. This was required because not much was known about the feasibility of intramuscular ACTH therapy twice a week in the year the study was started. After approval the study was continued and in total 20 patients were included.

## Results

**Fig 1:**
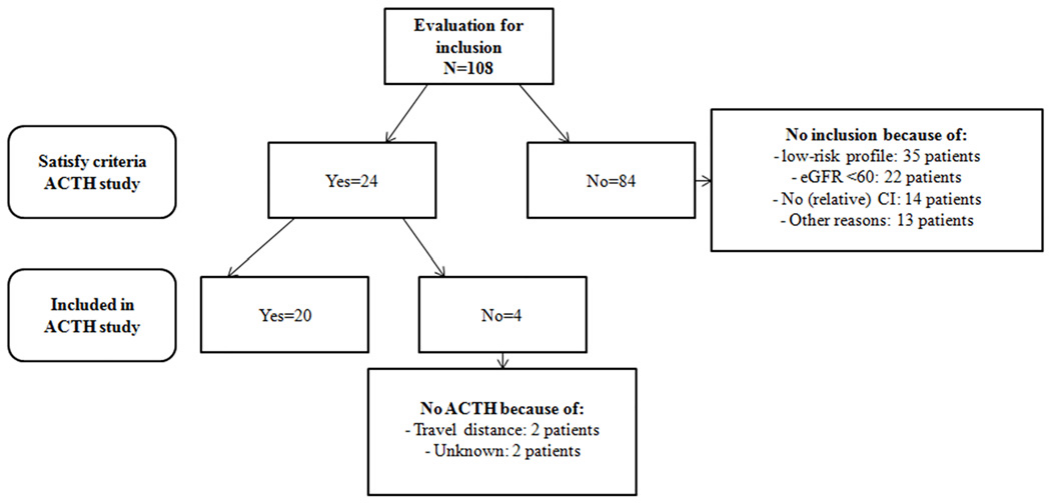
Flowchart (algorithm) of patient inclusion. Legend: eGFR = estimated glomerular filtration rate (by MDRD 4 equation), CI = contraindications, ACTH = synthetic ACTH, NS = nephrotic syndrome.

From February 2008 till December 2010 20 patients were enrolled in the study. In total 108 patients were evaluated in our outpatient clinic during that period. Eventually 24 patients were eligible for participating in the study; of whom 4 patients were not included (Fig 1). The baseline characteristics of the 20 patients are shown in Table 1. In the ACTH treated group there were 2 patients with diabetes mellitus, the time lag between biopsy showing no diabetic changes and initiation of ACTH was respectively 58 and 68 months. All patients in the ACTH group received at least 4 months of a maximum dose of RAAS inhibition before study inclusion, except for one patient that was only treated for 2.5 months with RAAS inhibition. Seventeen patients (85%) completed treatment; one patient was withdrawn from the study after the first injection because of progressive renal failure, in two other patients ACTH was stopped because of side effects: in one patient after 14 injections because of severe dysregulation of her diabetes mellitus and in one patient after 38 injections because of a delirium and infection. The percentage of injections that was administered correctly during the complete treatment period was 97%. The mean self-reported impact/burden of the treatment in the group on a scale of 1 to 10 was 7. An overview of the reported adverse events is listed in Table 2. Overall 19 patients (95%) documented at least one or more adverse events. Dose reduction was necessary in 2 patients (10%). Five patients needed hospitalization because of: hyperglycemia in known type I diabetes mellitus (without ketoacidosis), hyperglycemia with severe dehydration, mood disorder and dyspnea, and severe hypokalemia (n = 2). Mood disorders, oedema, myalgia/arthralgia, sleeping disturbances, fever/ infection, flushing, erythema/local reaction and hyperpigmentation of the skin were among the most common reported adverse events.

**Table 1:** Baseline characteristics.

	ACTH group (n = 20)	Cyclophosphamide group (n = 20)	P-value
Gender (male/female)	15:5	16:4	1.0
Age (years)	54 ± 15	50 ± 13	0.43
Diabetes mellitus	2 (10%)	1 (5%)	1.0
Serum creatinine (μmol/l)	104 [90–113]	97 [85–119]	0.81
Serum albumin (g/l)	22 ± 6.9	22 ± 5.5	0.96
eGFR (ml/min/1.73m²)	62 [52–76]	71 [53–76]	0.58
PCR (g/10 mmol Cr)	8.7 [4.3–11]	9.4 [6.7–12.2]	0.19
β2m excretion (ng/min)	2032 [685–3816]	3732 [1581–9673]	0.06
α1m excretion (μg/min)	55 [39–83]	72 [53–100]	0.12
Previous IS treatment	4 (20%)*	2 (10%)**	0.66
Interval Biopsy-T0	11 [3–56]	8 [6–13]	0.79
Follow-up (months)	46 ± 13	108 ± 45	<0.001

Values are expressed as number (percent), means ± SD and median [interquartile ranges]. ACTH = synthetic ACTH, eGFR = estimated glomerular filtration rate (by MDRD 4 equation), PCR = protein:creatinine ratio, β2m excretion = urinary beta2-microglobulin excretion, α1m excretion = urinary alfa1-microglobulin excretion, min = minute, T0 = start of ACTH treatment.

* The interval between the end of previous treatment and start of the current treatment was respectively 20, 38, 80 and 162 months in the ACTH group.

** In the CP treated group this interval was 27 months in one patient and 257 months in the other patient.

**Table 2:** Adverse events with ACTH treatment.

	No. of events	No. of patients (%)
Mood disorders/agitation	8	8 (40%)
Increasing oedema	12	12 (60%)
Myalgia/arthralgia	7	7 (35%)
Sleeping disturbances	10	10 (50%)
Fever/infection	9	8 (40%)
Flushing	7	7 (35%)
Hyperpigmentation skin	8	8 (40%)
Hypokalemia	7	3 (15%)
Erythema/local reaction	6	6 (30%)
Hypertension	5	5 (25%)
Weigth gain	6	6 (30%)
Acne	4	4 (20%)
Hyperglycemia	4	4 (20%)
Hair growth/hirsutism	4	4 (20%)
Cushingoid face	4	4 (20%)
Leukopenia	1	1 (5%)
Other	43	16 (80%)
**Overall**		
No. of patients with 1 or more AE		19 (95%)
No. of patients needing dose decrease		2 (10%)
No. of SAE (hospitalizations)	5 (25%)	5 (25%)

AE = adverse events, SAE = serious adverse events.

**Fig 2:**
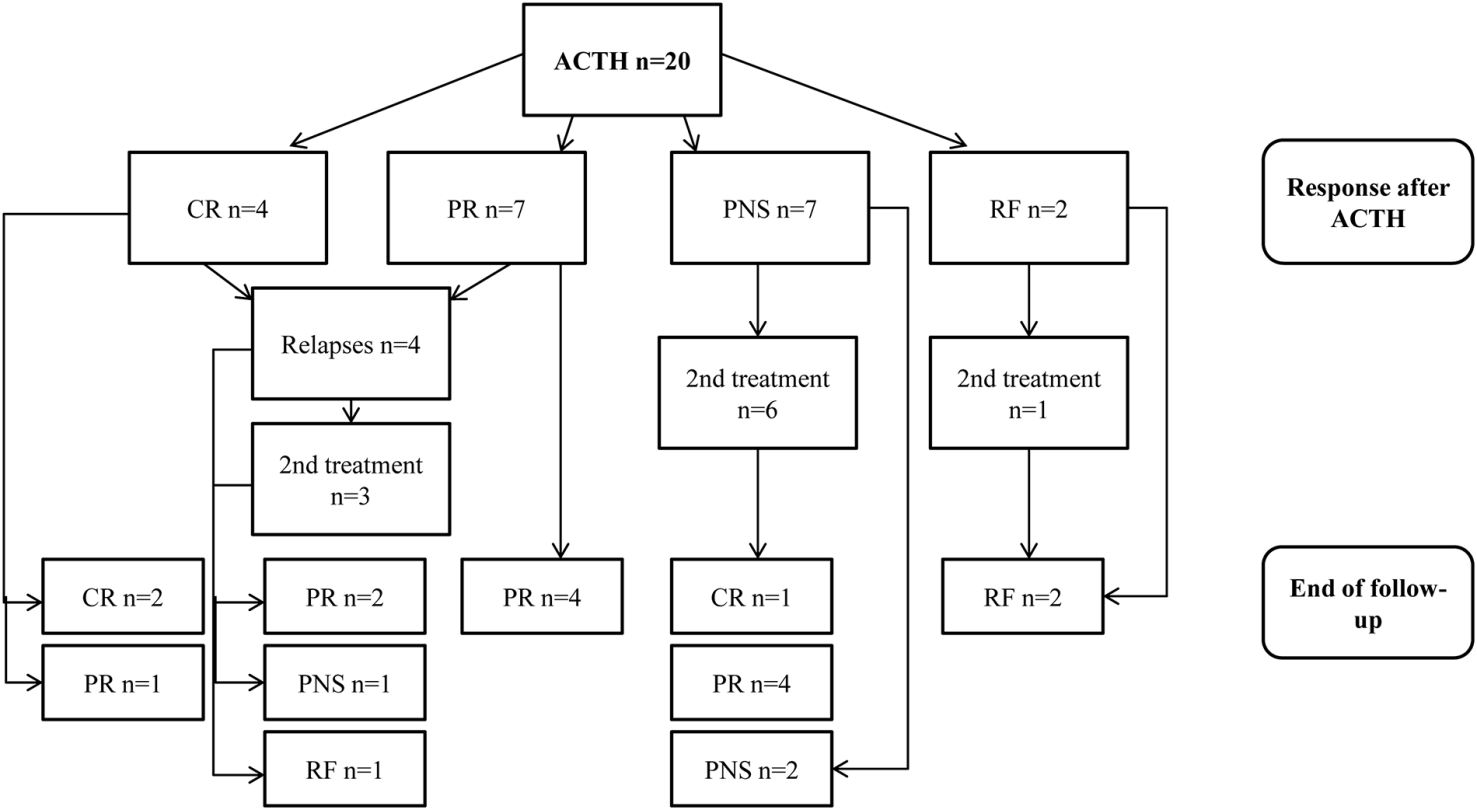
Flowchart of treatment and events during follow-up in the ACTH group. Legend: ACTH = synthetic ACTH, CR = complete remission, PR = partial remission, PNS = persisting nephrotic syndrome, RF = renal failure, 2^nd^ treatment = alternative immunosuppressive treatment

Proteinuria decreased from 8.7 [IQR 4.3–11.1] at baseline to 2.0 g/10 mmol creatinine [IQR 0.8–4.5] at end of treatment, a reduction of 64% [IQR 41–91]. Meanwhile GFR increased from 62 [IQR 52–76] at baseline to 72 ml/min/1.73m^2^ [IQR 60–81] at end of therapy. The outcomes after treatment with ACTH are depicted in Fig 2. Overall 11 patients (55%) developed a remission after treatment with ACTH; a complete remission in 4 patients and a partial remission in 7 patients (Table 3). Per-protocol analysis would increase the probability of response to 65% (11 of the 17 patients who completed treatment). Time to remission was 5 months [IQR 5–12]. Of these 11 patients four persons (36%) developed a relapse during follow-up. Time to relapse was 16 ±8 months. In total ten patients (50%) needed additional treatment because of renal function deterioration (n = 3), persisting severe nephrotic syndrome (n = 5) and relapse (n = 2). One of the patients with a persisting nephrotic syndrome was previously treated with CP, none of the patients with renal deterioration was previously treated with another immunosuppressive agent. Of these 10 patients one patient was withdrawn from the study after the first injection because of progressive renal failure, and the one patient who had to stop because of severe dysregulation of her diabetes mellitus was treated with CP because of persisting nephrotic syndrome. At the end of follow-up 14 patients (70%) were in remission, which was complete in three of them, and partial in 11.

**Table 3:** Clinical outcomes ACTH group vs. Cyclophosphamide group.

	ACTH group	CP group	p-value
**Remission after first therapy**	11/20 (55%)	19/20 (95%)	0.01
Complete remission	4/11	13/19	
Partial remission	7/11	6/19	
**Time to remission (m)**	5 [5–12]	6 [3–8]	0.40
**Relapse**	4/11 (36%)	7/19 (37%)*	
**Time to relapse (m)**	16 ± 8	86 ± 37	0.02
**Follow-up (months)**	46 ± 13	108 ± 45	<0.001
**Remission at end of follow-up**	14/20 (70%)	15/20 (75%)	1.00
Complete remission	3/14	6/15	
Partial remission	11/14	9/15	
Without additional treatment	7/14 (50%)	13/15 (87%)	0.05
**Additional treatment**	10/20 (50%)	4/20 (20%)	0.10
Relapse	2/10	3/4	
Progressive disease	8/10	1/4	

Values are expressed as number (percent), median [interquartile ranges] and mean ± SD. ACTH = synthetic ACTH, CP = cyclophosphamide, m = months. Progressive disease = renal function deterioration and persisting nephrotic syndrome.

*The relapse rate after 46 months is 5.3% (1/19 patients) in the CP group.

### ACTH vs. Cyclophosphamide

For comparison we selected 20 historical controls treated with CP (Table 1); well matched for baseline characteristics. In the CP group 18 patients (90%) completed treatment. Reasons for withdrawal were severe leucopenia in one patient, for which CP was switched after one month to azathioprine and prednisone during one year and in one patient treatment was discontinued after 7 months for an unknown reason. Dose reduction because of side effects was necessary in 7 (35%) patients. This was not significantly different from the ACTH group. In the CP group 11 patients (55%) reported one or more adverse events; 7 patients developed bone marrow depression, 5 patients suffered from an infection and 1 patient had a hyperglycemia.

**Fig 3:**
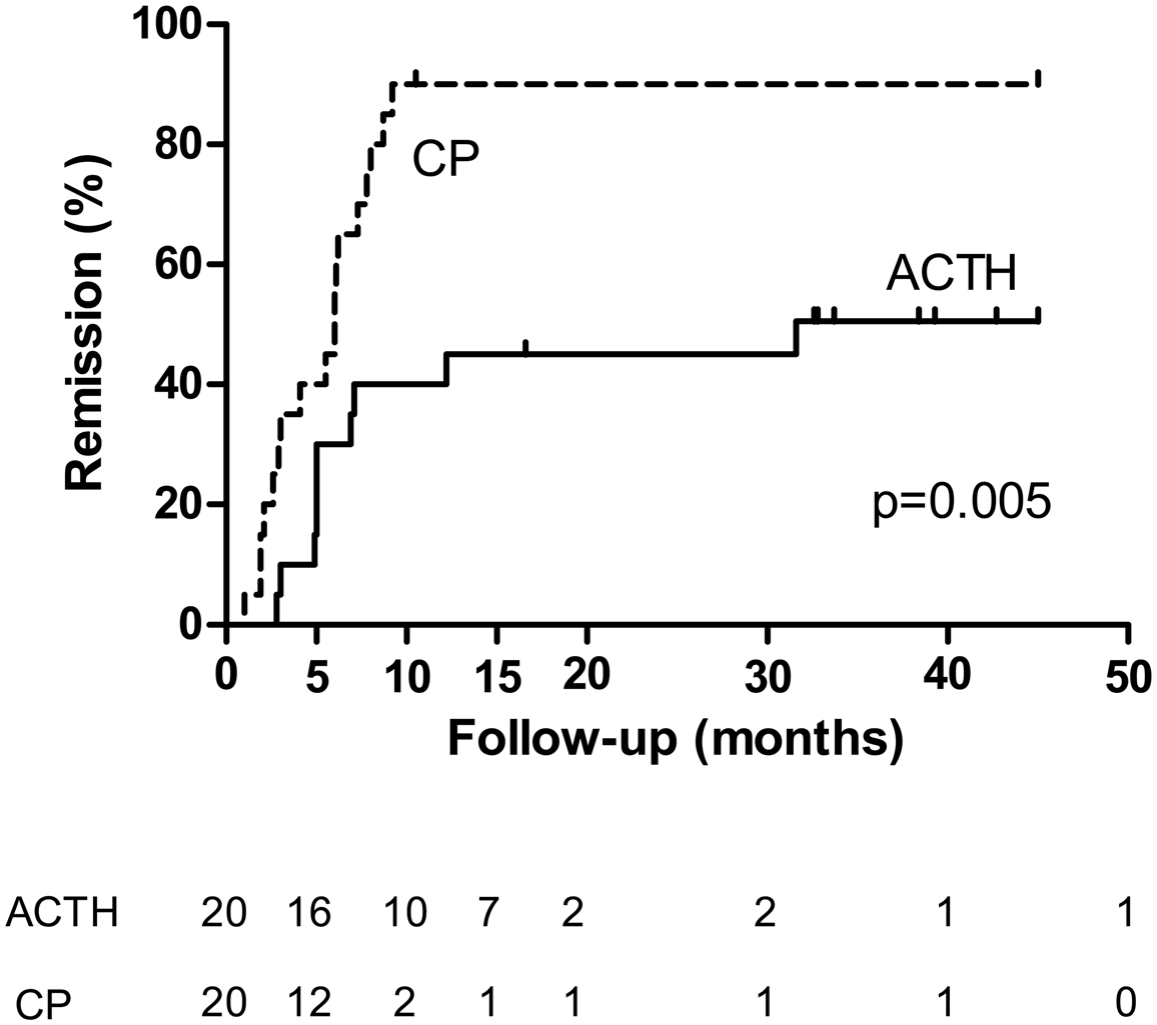
Kaplan-Meier plot for cumulative incidence of remission. Legend: Number of patients at risk for a remission at each time point are given below the figure. Log-rank test p = 0.005. ACTH = synthetic ACTH, CP = cyclophosphamide.

Proteinuria decreased from 9.4 [IQR 6.7–12.2] at baseline to 0.6 g/10 mmol creatinine [IQR 0.3–1.0] at end of treatment, a reduction of 92% [IQR 85–97] with stable eGFR (71 [IQR 53–76] at baseline to 71 ml/min/1.73 m² [IQR 55–77] at end of treatment). The decrease of proteinuria was significantly higher during treatment with cyclophosphamide compared with ACTH (92% versus 64%, p = 0.004). After treatment with CP, significantly more patients developed a remission (Table 3). Both patients who had to discontinue treatment with CP entered remission. Time to remission was not significantly different between the two groups. The cumulative incidence of remission and time to remission are depicted in Fig 3.

**Fig 4:**
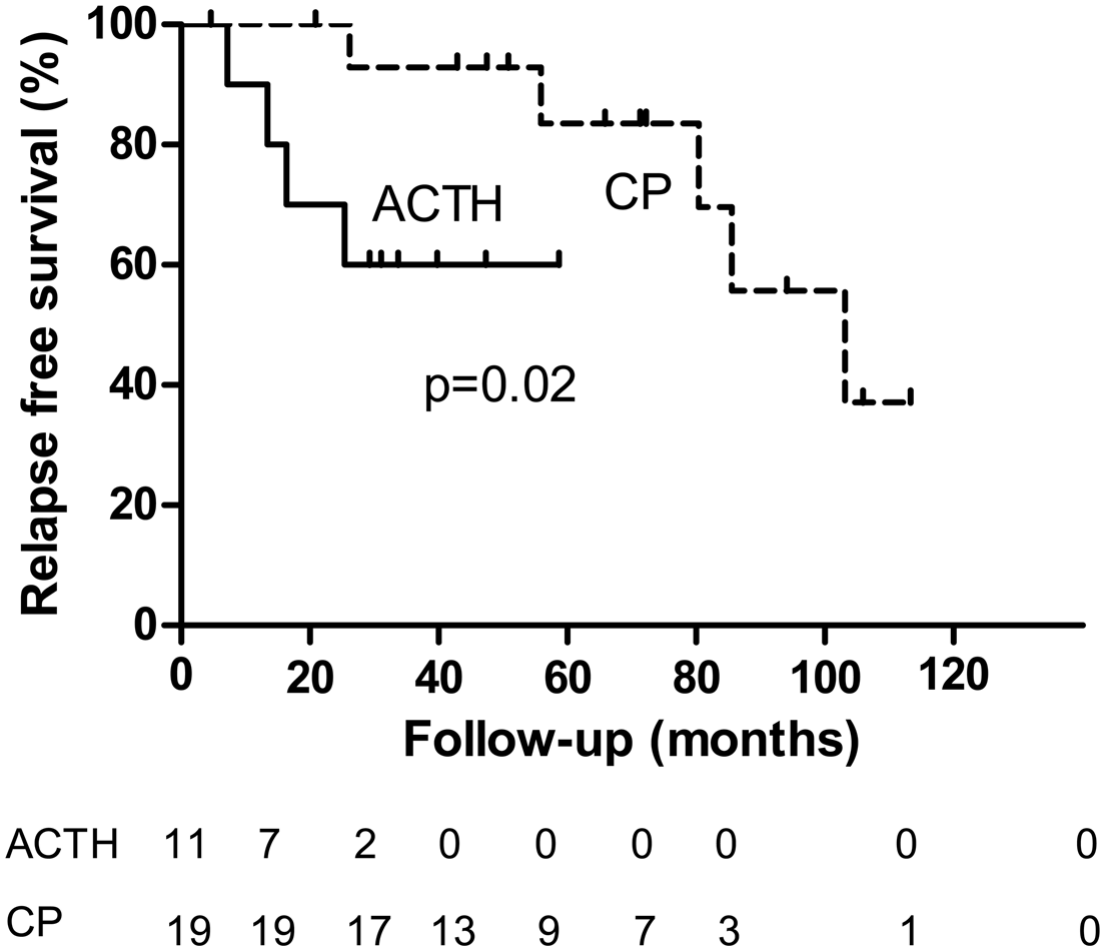
Kaplan-Meier plot for relapse free survival. Legend: Number of patients at each time point are given below the figure. Log-rank test p = 0.020. ACTH = ACTH, CP = cyclophosphamide.

Relapses occurred in four of the 11 (36%) patients in the ACTH group and in seven of the 19 (37%) patients in the CP group. Of note, the duration of follow-up was much longer in the CP group. A log-rank test showed a shorter relapse free survival for patients in the ACTH group (p = 0.02), depicted in Fig 4. In the CP group less patients needed additional immunosuppressive treatment during follow-up, although this was not significant (Table 3). Results and conclusions were similar when comparing outcome in ACTH treated patients with outcome in all CP treated patients (S2 Table).

## Discussion

Our data show that treatment with intramuscular injections of synthetic ACTH twice a week in an outpatient setting is feasible. The mean self-reported impact/burden of therapy was considerably, with a score of 7 on a scale of 1 (very unsatisfied) to 10 (very satisfied). Although this is not a validated measuring instrument, this reflects the substantial impact of the treatment for patients, something that has not previously been mentioned in literature. Besides overall response rate was moderate and we noted many adverse events.

In our study, all but one patient documented at least one adverse event. Five patients needed hospitalization because of serious adverse events. Treatment was discontinued because of side effects in two patients (10%). Thus far the documented adverse events in other studies were considered as relatively mild. Still, in five out of 51 patients (10%) ACTH was withdrawn. [11, 13, 14]Thus, although most adverse events are mild, severe adverse events do occur during treatment with (synthetic) ACTH and sometimes force discontinuation of treatment.

Our data suggest that treatment with synthetic ACTH is less effective than CP in inducing a remission (55% vs. 95%) in high risk patients with iMN. At baseline urinary β2m excretion in the CP treated group was higher than in the ACTH treated group. It is possible that CP treated patients were therefore even more vulnerable for progression to kidney failure. Despite this difference however treatment with synthetic ACTH was less effective than CP in inducing a remission and also more rapid relapses were noted in the ACTH treated group. At the end of follow-up the rate of remission was equal in the ACTH and CP treated groups (70% and 75% respectively), although patients in the ACTH group had needed more often additional therapy. Moreover, no major differences between short time side effects were observed between the two groups. Of note, adverse events were not recorded in the same way in the historical controls as in the study population, the latter being actively questioned about side effects.

This is the first prospective study of synthetic ACTH in a cohort of patients with iMN at high risk for end stage renal disease. Remission rates in other studies may have been inflated by spontaneous remissions, which occur in up to half of unselected patients with iMN. [3, 10, 11, 20]

Indeed several authors have reported benefit of treatment with ACTH in patients with iMN and a high risk profile.[12–14] However another formula of ACTH, i.e. H.P. Acthar^®^ gel was used in these studies. Bomback et al. report on 11 patients with iMN, who previously had failed a mean of 2.4 therapies.[12] Treatment with various doses of Acthar^®^ gel resulted in remission of proteinuria in 9 patients, with complete remission in three of them. In a more recent prospective study, 2 out of 5 patients with treatment resistant MN achieved a remission of proteinuria after treatment with Acthar^®^ gel.[13] Hladunewich et al. treated twenty patients with iMN (seven of them with partial response to other immunosuppressive regimens or significant side effects during previous treatment) with 40 or 80 IU Acthar^®^ gel twice weekly during 12 weeks and in a subgroup (5 patients) for an additional 90 days after dose increase. [14] After 1 year of follow-up 2 patients achieved a complete remission (10%) and 10 patients achieved a partial remission (50%). Definition of high risk in these above mentioned studies was different from our criteria for high risk. This makes the comparison of our treated cohort with the abovementioned patients difficult. We also cannot exclude differences in efficacy between synthetic ACTH and Acthar^®^ gel, as there might be differences in active ingredients (see below) and we don’t know if the doses of synthetic ACTH used and Acthar^®^ gel are comparable.

The particular strength of our study is that this is the first study in which ACTH was tested in a homogeneous high risk group. Another strength is the long duration of follow up. The main limitation of our study is its small size and the use of a historical control group. Therefore the conclusions should be regarded with caution. Still, we feel that our data clearly suggest that synthetic ACTH is less effective than cyclophosphamide in high risk patients with idiopathic membranous nephropathy.

Anti-PLA2R measurement was not available at the inclusion period of our study. Hladunewich et al. previously showed that treatment with Acthar^®^ gel induced clearing of antibodies prior to or in parallel with decrease in proteinuria in some, but not all anti-PLA2R positive patients. As we previously showed, that anti-PLA2R antibodies rapidly disappeared after treatment with cyclophosphamide in the majority of patients [21] it would have been interesting to compare antibody response between our ACTH treated and cyclophosphamide treated groups.

It remains unknown how ACTH might induce clinical remission in patients with iMN, although many mechanisms of actions have been proposed. Synthetic ACTH is a long-chain polypeptide composed of the first 24 of the 39 amino acids contained in the naturally occurring ACTH (corticotrophin) molecule. The molecule can be cleaved, with the first 13 amino acids forming α-melanocyte-stimulating hormone (α-MSH). (www.uniprot.org/uniprot/P01889) Both peptides interact with melanocortin receptors (MCR, types 1–5 identified, with different tissue distributions). It is unlikely that the antiproteinuric effect of ACTH is solely related to increased release of cortisol and steroidogenesis via MCR2. [22] Other potential working mechanisms of ACTH are: activation of MCR1 on podocytes [23], anti-inflammatory actions in the kidney through MCR 2 interaction, direct MCR-mediated systemic immunomodulation and anti-inflammatory effects on peripheral blood leukocytes (MCR 1, 3 and 5), kidney protection secondary to correction of dyslipidemia mediated by the MCR on hepatic cells (MCR 1, 5) and renal protection via neurogenic anti-inflammatory effects mediated by MCR’s expressed in the central nervous system (MCR 3, 4). [24] Acthar ^®^ gel is obtained from processing porcine pituitary glands. There is evidence that the synthetic analogues are different from natural occurring ACTH. For example, the β-cell tropic and insulinotropic effects of the endogeneous hormone resides in the C-terminus of ACTH (fragment 18–39)[25], which is omitted from the synthetic analogue but is retained in ACTH gel. Moreover, other active ingredients derived from pro-opiomelanocortin (POMC), probably include additional melanocortin peptides such as β- and γ-MSH. [24] It cannot be excluded that Acthar ^®^ gel has an additional mechanism of action compared to synthetic ACTH. Direct comparisons between treatment with synthetic ACTH and Acthar ^®^ are needed.

In conclusion, treatment with intramuscular injections of synthetic ACTH is feasible. Our data suggest that synthetic ACTH is less effective than CP in inducing a remission in high risk patients with iMN. The use of synthetic ACTH was also associated with many adverse events. Therefore, we advise against synthetic ACTH as standard treatment in membranous nephropathy.

## Supporting Information

S1 Protocol Protocol-CMO.doc. (PDF)

S2 Protocol Protocol_englishtranslation.doc. (DOCX)

S1 Table Dose and amount of synthetic ACTH injections. (DOCX)

S2 Table Outcomes ACTH treated patients vs. all cyclophosphamide treated patients. (DOCX)

S1 TREND Checklist trendstatement_trend_checklist part 1.pdf. (PDF)

S2 TREND Checklist trendstatement_trend_checklist part 2.pdf. (PDF)

S3 TREND Checklist trendstatement_trend_checklist part 3.pdf. (PDF)
