# Supplementary material for: Synthetic ACTH in High Risk Patients with Idiopathic Membranous Nephropathy: A Prospective, Open Label Cohort Study
Source: PLoS One. 2015 Nov 12;10(11):e0142033. doi: 10.1371/journal.pone.0142033 (PMC4642982; doi:10.1371/journal.pone.0142033)
Supplement: S1 Protocol — (PDF) [file pone.0142033.s001.pdf]

Protocol:

Synthetisch adrenocorticotroop hormoon (ACTH) bij de behandeling van patiënten met een membraneuze glomerulonefritis en een hoog risico op nierfalen. Een pilot-studie.

Versie 1 dd. 14 maart 2008

Studie coördinatoren:
Drs.J.M.Hofstra
Prof.Dr.J.F.M.Wetzels
Afdeling Nierziekten 464
UMC St Radboud
Postbus 9101
6500 HB Nijmegen
T: 024 3614761
F: 024 3540022

Inhoudsopgave

Algemene informatie …...……………………………………………………….. 2
Achtergrondinformatie ……………………………………………………………… 3
Doelstellingen ..……...…………………………………………………….. 6
Studieopzet ………………………………………………………………… 7
Eindpunten ………………………………………………………………. 7
Patiënten ………………………………………………………………… . 7
Behandelschema ………………………………………………………… 8
Follow-up ………………………………………………………………… 8
Stop behandeling ………………………………………………………. 9
Beoordeling van werkzaamheid ……………………………………….. 9
Beoordeling van veiligheid …………………………………………….. 10
(Statistische) analyse …………………………………………………… 11
Kwaliteitsbeheersing en borging ……………………………………………….. 12
Inzage in brongegevens/brondocumenten ……………………………… 12
Ethische aspecten ……………………………………………………………. 13
Verwerking en vastlegging van gegevens ……………………………………… 14
Financiering en verzekering ……………………………………………………….. 15
Administratie en publicatiebeleid ……………………………………………. 16
Referenties ………………………………………………………….……… 17
Samenvatting Protocol ……………………………………………………… 20

Bijlagen binnen protocol

1: procedure proteïnuriemeting ……...…………………………………………..23
2: follow-up schema ……………………………………………………………….21
3: formulieren rapportage Adverse Event (AE) en Serious Adverse Event (SAE)……...25
4: Case Record Form ……………………………………………eigen pagina nummering 1-24

Bijlagen buiten protocol (nummering volgens eisen METC):

D2: product informatie onderzoeksproduct (incl. SPC's en gedetailleerd behandelschema)
D3: voorbeeldetiketten studiemedicatie
E1: patiëntinformatie
E2: verklaring van deelname / toestemmingsformulier

Algemene informatie:

Titel: Synthetisch adrenocorticotroop hormoon (ACTH) bij de behandeling van patiënten met een membraneuze glomerulonefritis en een hoog risico op nierfalen. Een pilot-studie.

Engelstalig: Treatment with synthetic adrenocorticotropic hormone (ACTH) in patients with membranous nephropathy and high risk for renal failure. A pilot study.

EudraCT-nummer: 2008-001647-19

Protocolnummer: 2008.1

Datum protocol: versie 14-3-2008

Verrichter/sponsor: Universitair Medisch Centrum St Radboud
Prof.Dr.J.F.M.Wetzels, internist-nefroloog
Afd. Nierziekten 464
Postbus 9101
6500 HB Nijmegen
024 3614761

Monitor: M.van Helden
Research-verpleegkundige
Afd. Nierziekten UMC St Radboud

Onderzoeker: Drs.J.M.Hofstra, internist in opleiding

Onderzoekslocatie: Afd.Nierziekten 464
UMC St Radboud
Geert Grooteplein 8
6525 GA Nijmegen
024 3614761

Onafhankelijk arts: Dr.G.Vervoort, internist-nefroloog
Afd.Algemeen Interne Geneeskunde en
Afd. Nierziekten
UMC St Radboud

Betrokken afdelingen:
Apotheek/Klinische Farmacie 864
UMC St Radboud
Geert Grooteplein 8
6525 GA Nijmegen
024 3614761

Klinisch Chemisch Laboraorium 441
UMC St Radboud
Geert Grooteplein 8
6525 GA Nijmegen
024 3614761

Achtergrondinformatie:

Idiopathische membraneuze glomerulonefritis (iMGN) is de meest voorkomende oorzaak van het nefrotisch syndroom op volwassen leeftijd, met in Nederland een incidentie van 10/miljoen/jaar.[1] In de literatuur worden nogal wisselende gegevens gerapporteerd over het natuurlijk beloop van de aandoening. Dit wordt ten dele verklaard door de heterogeniteit van de onderzochte patiëntenpopulaties o.a. wat betreft de verdeling naar leeftijd en geslacht, de nierfunctie, de mate van proteïnurie en de duur van de follow-up. De resultaten van lange termijn studies (met een follow-up van 9-14 jaar) kunnen globaal als volgt worden samengevat: indien er niet wordt behandeld, ontwikkelt 14-56% van de patiënten een spontane remissie, terwijl 34-62% van de patiënten een nierinsufficiëntie ontwikkelt.[2;3] Na 10 jaar follow-up wordt een persisterend nefrotisch syndroom slechts bij 4-9% van de patiënten gezien.

Over de behandeling van patiënten met een membraneuze glomerulonefritis bestaat geen eenstemmigheid.[4;5] De resultaten van behandeling met alleen corticosteroïden zijn teleurstellend.[6-8] Behandeling met chloorambucil dan wel cyclofosfamide met corticosteroïden laat wel gunstige effecten zien met snellere en frequentere remissie van het nefrotisch syndroom en een betere preservatie van de nierfunctie.[3;9;10] In onze patiëntenpopulatie bleek behandeling met een combinatie van cyclofosfamide en prednison effectiever dan de combinatie van chloorambucil en prednison. Met name was er tijdens de behandeling met cyclofosfamide vaker sprake van een remissie van de proteïnurie (15/17 vs 5/15 patiënten) en een langduriger verbetering van de nierfunctie met minder bijwerkingen.[9]
Alternatieve behandelingen zijn slechts beperkt onderzocht. Behandeling met cyclosporine blijkt minder effectief met het optreden van recidief proteïnurie na stoppen van de therapie.[11-13] Tevens is het middel door zijn nefrotoxiciteit maar beperkt toepasbaar bij patiënten met een gestoorde nierfunctie.[12]
Azathioprine is niet effectief en de behandeling gaat frequent gepaard met bijwerkingen.[14-16] Mycofenolaat mofetil (MMF) lijkt niet effectiever in het induceren van een remissie dan de alkylerende middelen en in onze patiëntpopulatie zagen we een hoog aantal recidieven binnen 2 jaar.[17;18] In recente studies bleek anti-B-cel therapie (rituximab) effectief voor een deel van de patiënten, terwijl andere patiënten geen respons vertoonden.[19;20]

Gezien het natuurlijke beloop van de ziekte MGN, waarbij in ca. 50% van de patiënten een spontane remissie optreedt, lijken er, gelet op de toxische effecten van immunosuppressieve therapie onvoldoende argumenten om alle patiënten met een MGN met cytotoxische therapie te behandelen. Om deze reden is er uitvoerig gezocht naar factoren die het optreden van nierinsufficiëntie voorspellen, met de bedoeling patiënten met een slechte prognose te identificeren en alleen deze patiënten te behandelen.

In het verleden werd gewezen op de voorspellende waarde van de hoogte en duur van de proteïnurie.[21;22] In de afgelopen 15 jaar hebben wij patiënten met een MGN en normale nierfunctie prospectief vervolgd. Wij vonden dat de uitscheiding in de urine van IgG en ß2-microglobuline het risico op nierfunctieverlies kon voorspellen met een hoge sensitiviteit en specificiteit.[23] De drempelwaarden voor excretie van ß2-microglobuline en IgG waren hierbij gelegd op respectievelijk 500 ng/min en 250 mg/dag. Deze bevindingen werden bevestigd door Bazzi et al, die α1-microglobuline (i.p.v. ß2-microglobuline) en IgG gebruikten als voorspellende markers.[24] In een recente studie hebben wij de voorspellende waarde van ß2-microglobuline en IgG volgens de gevonden drempelwaardes gevalideerd, waarbij de combinatie van deze markers toekomstig nierfunctieverlies voorspelt met een sensitiviteit en specificiteit van respectievelijk 83% en 97%.[25] β2-microglobuline is hierbij de belangrijkste voorspeller voor het optreden van nierfunctieverlies.

Algemeen aanvaard is dat patiënten met een MGN met nierfunctieverlies (serum creatinine > 135 μmol/l) behandeld dienen te worden, gezien de te verwachten progressie tot een terminale nierinsufficiëntie. Op basis van de gevalideerde resultaten van onze voorspellende markers, delen wij momenteel patiënten met een MGN met nefrotisch syndroom en een normale nierfunctie in in een hoog en laag risico groep met behulp van de ß2-microglobuline excretie in de urine. Patiënten met een hoog risico op nierfunctieverlies worden zodra het serum kreatinine gaat stijgen gedurende een jaar behandeld met cyclofosfamide en prednison, waarbij bij een kinderwens cyclofosfamide na 3 maanden vervangen wordt door azathioprine.

Eerder publiceerden wij lange termijn-resultaten van deze behandeling.[26] De behandeling met cyclofosfamide kent vanwege de toxiciteit van het middel meerdere nadelen. Frequent treden bijwerkingen op, zoals (pan)cytopenie, die dosisreductie noodzakelijk maken. Daarnaast bestaat er een verhoogde kans op ernstige infectieuze complicaties en gaat lange termijn behandeling gepaard met een verhoogd risico op (blaas)maligniteiten.[27] Als laatste, maar niet minder belangrijk, kan cyclofosfamide leiden tot infertiliteit. De kans op infertiliteit neemt toe met de cumulatieve dosis, reden om bij patiënten met een kinderwens na 3 maanden over te gaan op het minder effectieve azathioprine.

Gezien de toxische effecten van cyclofosfamide wordt er gezocht naar een alternatieve behandeling. Berg et al. zagen in een studie naar de effecten van langwerkend synthetisch ACTH (adrenocorticotroop hormoon) op het lipiden profiel van patiënten met een nefrotisch syndroom bij MGN een onverwacht gunstig effect op de afname van de proteinurie.[28] Tijdens de behandeling met ACTH verminderde de proteïnurie met 90% en bereikten alle vijf patiënten een remissie. Dit effect werd in een tweede studie bevestigd.[29] In een recente gerandomiseerde pilotstudie van Ponticelli et al. in patiënten met MGN en een nefrotisch syndroom, was synthetisch ACTH even effectief als behandeling met steroïden en een alkylerend middel (chloorambucil of cyclofosfamide).[30] In beide groepen van 16 patiënten traden evenveel remissies op (resp. 87% en 75%, verschil in het voordeel van ACTH niet significant), waarbij het van belang is te weten dat de patiënten in deze studie niet geselecteerd waren op een hoog risico voor toekomstig nierfunctieverlies. De bijwerkingen van synthetisch ACTH waren in deze studies mild en reversibel, en gerelateerd aan mineralo- en glucocorticoïde effecten. Optredende bijwerkingen waren onder andere glucose-intolerantie, milde Cushingoïde trekken, vochtretentie, slaapstoornissen, hyperpigmentatie van de huid en diarree. Overgevoeligheidsreacties op synthetisch ACTH werden in deze studies niet gezien.

De exacte mechanismes achter het effect van ACTH op proteïnurie en glomerulaire functie is niet duidelijk. ACTH is een hypofyse hormoon dat de bijnieren stimuleert tot de productie van met name glucocorticoïden (cortisol). Het is echter onwaarschijnlijk dat het anti-proteïnurische effect van synthetisch ACTH volledig berust op de toegenomen endogene cortisol productie. De gebruikte dosering synthetisch ACTH in de studies van Berg et al. en Ponticelli et al. (2/wk 1mg) correspondeert met ongeveer 300 mg cortisol per week, hetgeen ruwweg het equivalent is van 10-15 mg prednison per dag.[31] In eerdere studies bleek monotherapie met corticosteroïden in veel hogere doseringen niet effectief in patiënten met MGN.[5-7] Bij de ACTH-geïnduceerde cortisolsynthese wordt een grote hoeveelheid endogeen cholesterol verbruikt, hetgeen de gunstige werking van ACTH op het lipidenprofiel verklaart. Hoewel de hyperlipidemie waarmee het nefrotisch syndroom gepaard gaat mogelijk gekoppeld is aan de prognose van de nierziekte, is het niet waarschijnlijk dat de verbetering van het lipidenprofiel het primaire werkingsmechanisme is.[32] Verbetering van het lipiden profiel wordt namelijk ook met de huidige behandeling met HMG- CoA reductase remmers bereikt.
Naast het effect op de cortisolsynthese stimuleert ACTH in mindere mate de productie van mineralocorticoïden en androgene steroïden.

Ondanks het feit dat het werkingsmechanisme van ACTH bij nefrotisch syndroom nog niet ontrafeld is, biedt de behandeling duidelijke voordelen. Het gebruik van hoge dosis steroïden en het cytotoxische cyclofosfamide – en dus de daarmee gepaard gaande bijwerkingen- wordt

vermeden. Hoewel er nog geen data zijn over de lange termijn resultaten en de eventuele kansen op een recidief van de ziekte na een door ACTH-geinduceerde remissie, zou alleen al het uitstellen van behandeling met alkylerende middelen voor jonge patiënten met een kinderwens een voordeel zijn. Mogelijk nadeel zou de praktische haalbaarheid van de behandeling kunnen zijn, daar 2 keer per week een intramusculaire injectie moet worden toegediend aan de patiënt. In verband met door de fabrikant beschreven overgevoeligheidsreacties moet de patiënt na toediening van een injectie 30 minuten geobserveerd worden. Hoewel dit voor de patiënt een (tijds-)belasting betekent, menen wij dat dit opweegt tegen het feit dat de alkylerende middelen vermeden worden.

Het doel van de huidige studie is te beoordelen of langwerkend synthetisch ACTH (tetracosactide hexaacetate/ Synacthen Depot®) een mogelijk alternatief is voor behandeling met cyclofosfamide en prednison bij patiënten met een MGN met normale nierfunctie, maar met een hoog risico op nierfunctieverlies. Hierbij willen we die patiënten includeren bij wie een relatieve contra-indicatie voor behandeling met cyclofosfamide bestaat. Het gaat hierbij om jonge patiënten met een kinderwens, patiënten ouder dan 60 jaar bij wie het risico op complicaties van de behandeling verhoogd is, patiënten die in het verleden reeds behandeld zijn met cyclofosfamide of patiënten met een intolerantie voor cyclofosfamide.

Het huidige onderzoek betreft een pilotstudie waarin aanvankelijk 6 patiënten behandeld zullen worden met Synacthen Depot. Er zal een behandelschema worden gebruikt, zoals dat in de groep van Berg en Arnadottir is ontwikkeld naar aanleiding van hun eerste 2 studies.[33] In tegenstelling tot de schema’s die in de tot nu toe gepubliceerde studies werden gebruikt, wordt de dosering in dit schema niet alleen opgebouwd, maar aan het eind van de periode ook afgebouwd. Dit om het theoretische risico van een relatieve bijnierschorsinsufficiëntie na langdurige behandeling te vermijden. Na een opbouwperiode van 2 maanden wordt gedurende 4 maanden 2 keer per week 1 mg Synacthen Depot toegediend als intramusculaire injectie door een arts of verpleegkundige/ doktersassistent. Hierna wordt deze dosering in een periode van 3 maanden geleidelijk afgebouwd. (Voor het exacte behandelschema en uitgebreide productbeschrijving van Synacthen Depot zie pagina 8 en bijlage D2).
Bij deze 6 patiënten zal met name gekeken worden of de behandeling met Synacthen Depot praktisch en logistiek uitvoerbaar is. Indien dit het geval is, zullen nog 14 patiënten volgens hetzelfde protocol behandeld worden, waarna de gegevens van de totale groep van 20 patiënten geanalyseerd zullen worden wat betreft de behandelresultaten.

NB: Voor patiënten bij wie tijdens de studie een stijging van het serum kreatinine optreedt van meer dan 25% of bij wie het serum kreatinine een waarde > 135 µmol/l bereikt, geldt dat de behandeling in het kader van de studie gestopt wordt en dat de standaard behandeling wordt gestart. De standaard behandeling omvat behandeling met cyclofosfamide en prednison gedurende 1 jaar; indien een kinderwens bestaat, wordt cyclofosfamide na 3 maanden vervangen door azathioprine.

Het onderzoek zal worden uitgevoerd in overeenstemming met het protocol, de regels van Good Clinical Practice en de relevante wettelijke vereisten.[34]

Doelstellingen:

Het doel van de huidige studie is te beoordelen of ACTH een mogelijk alternatief is voor behandeling met cyclofosfamide en prednison bij patiënten met een MGN met normale nierfunctie, maar met een hoog risico op nierfunctieverlies en hoog risico op complicaties van de standaardbehandeling met cyclofosfamide.

Reden voor de studie is het feit dat alternatieve behandeling gewenst is. De huidige behandeling met cyclofosfamide en prednison kan leiden tot ernstige bijwerkingen. Daarnaast is de behandeling met name bij jonge mensen met een kinderwens niet goed toepasbaar in verband met het risico op infertiliteit bij het gebruik van cyclofosfamide.

Primair doel:
Evalueren van de haalbaarheid en praktische uitvoerbaarheid van therapie met synthetisch ACTH bij 6 patiënten met MGN en een hoog risico op nierfalen.

De uitvoerbaarheid zal worden geëvalueerd aan de hand van het percentage injecties dat gedurende de behandeling op het juiste moment kon worden toegediend.

Secundaire doelen:

- Evalueren van de effectiviteit van ACTH bij de behandeling van membraneuze glomerulonefritis.

  Indien de therapie met synthetisch ACTH haalbaar en praktisch goed uitvoerbaar is gebleken (>85% van de injecties toegediend op het juiste moment), zullen 14 additionele patiënten in de studie worden geïncludeerd voor onderzoek naar de effectiviteit (totaal 20 patiënten).
  Indien bij de eerste 6 patiënten is gebleken dat de studie niet voldoende praktisch uitvoerbaar is, zullen geen extra patiënten worden geïncludeerd en zal de analyse naar de effectiviteit slechts de eerste 6 patiënten betreffen.

  De effectiviteit van de behandeling wordt geëvalueerd wat betreft:
  - het percentage bereikte remissies.
  - de verandering van de nierfunctie (kreatinine klaring volgens MDRD) in de tijd
  - de veiligheid van ACTH uitgedrukt in het aantal bijwerkingen.

- Inzicht verkrijgen in het werkingsmechanisme van ACTH bij membraneuze glomerulonefritis door nagaan van het effect van ACTH op de endogene cortisol/cortison productie.

Studieopzet

Het betreft een open, niet gecontroleerde pilot-studie.

Eindpunten:
Primair
Haalbaarheid van 2 keer per week toedienen van een intramusculaire injectie synthetisch ACTH gedurende 9 maanden; uitgedrukt in het percentage injecties dat gedurende de behandeling op het juiste tijdstip is toegediend. Het juiste tijdstip is hierbij gedefinieerd als de geplande datum ± 1 dag.

Secundair
- Door de patiënt ervaren belasting van de behandeling na 9 maanden: weergegeven op een schaal van 1 tot 10.
- Aantal patiënten dat de gehele behandeling van 9 maanden completeert.
- Aantal remissies, zowel compleet als partieel (proteïnurie resp. < 0.2 g/dg of <2.0 g/dg)
- Aantal complete remissies (proteïnurie < 0.2 g/dg) op tijdstippen 9 en 24 maanden
- Aantal partiële remissies (proteïnurie <2.0 g/dg) op tijdstippen 9 en 24 maanden
- Verandering van de proteïnurie (indien geen remissie bereikt) vanaf t= 0 tot zowel 9 als 24 maanden, uitgedrukt als percentage
- Verandering van nierfunctie (veranderde klaring) vanaf t= 0 tot zowel 9 als 24 maanden, uitgedrukt als percentage
- Aantal bijwerkingen.

Patiënten:

Aanvankelijk zullen 6 patiënten geïncludeerd worden. In 2^{e} instantie kunnen nog 14 patiënten worden geïncludeerd indien analyse van het primaire eindpunt (zie verder) heeft uitgewezen dat er sprake is van een goede haalbaarheid en praktische uitvoerbaarheid van de behandeling volgens het studieprotocol.

Patiënten kunnen worden geïncludeerd vanuit de polikliniek Nierziekten in het UMC St Radboud. Daarnaast bestaat er reeds lange tijd een samenwerkingsverband met nefrologen uit de regio, waarbij hen gevraagd zal worden geschikte patiënten voor de studie te verwijzen.

Inclusiecriteria:
- Leeftijd 18-75 jaar
- Idiopathische membraneuze glomerulonefritis, biopsie bewezen.
- Nefrotisch syndroom: proteïnurie ≥ 3.5 g/dag en serum albumine ≤ 30 g/l
- Normale of licht gestoorde nierfunctie (eGFR > 60 ml/min/1.73m^2, eGFR volgens MDRD formule)
- Hoog risico op nierfalen: β2-microglobuline excretie > 500 ng/min (volgens Proteïnuriemeting, zie bijlage 1)
- Relatieve contra-indicatie voor behandeling met cyclofosfamide:
    a. kinderwens
    b. hoge leeftijd (> 60 jaar)
    c. eerdere behandeling met cyclofosfamide
    d. intolerantie voor cyclofosfamide

Exclusiecriteria:

- Klinische, biochemische of histologische tekenen van een onderliggende systeemziekte.
- Een infectieziekte (inclusief latente tuberculose en/of latente amoebiasis)
- Actief ulcuslijden (maag/duodenum)
- Zwangerschap, borstvoeding, inadequate anticonceptie
- Klinische tekenen van niervenetrombose
- Astma en/ of bekende allergieën of hypersensitiviteitsreacties
- Allergische reactie op synthetisch ACTH in het verleden

Behandelschema:

- Synthetisch ACTH (tetracosactide hexaacetate/ Synacthen Depot) 1mg/ml voor intramusculaire injectie. Eén injectie á 1 ml bevat 1 mg tetracosactide. Maximale dosering 2 maal per week 1 mg, totale duur van de behandeling 9 maanden.

- Bij aanvang van de behandeling wordt de dosering opgebouwd van 1 injectie per week naar 2 injecties per week gedurende een periode van 2 maanden (8 weken). De injecties worden aldus gegeven op dag 0, 7, 14, 21, 28, 32, 37, 42, 46, 51 en 56. Hierna wordt gedurende 4 maanden (18 weken) doorgegaan met 2 injecties per week. In maand 7 wordt begonnen met een afbouwschema waarbij de dosering in 3 maanden tijd wordt afgebouwd tot stop. De injecties worden dan gegeven op dag 186, 191, 196, 200, 205, 210, 217, 224, 231, 238, 245, 259 en 273. Een injectie mag maximaal 1 dag voor of na de geplande dag worden toegediend. Totaal aantal injecties per patiënt: 59
(zie ook bijlage D2)

- De injecties worden intramusculair toegediend door een verpleegkundige of arts. Na de injectie wordt de patiënt gedurende 30 minuten geobserveerd vanwege mogelijke overgevoeligheidsreacties. De 1e injectie vindt per definitie plaats in het UMC St Radboud.
- Eventueel kan na overleg verdere injecties worden toegediend op de dialyseafdeling in het regionale ziekenhuis na persoonlijke instructie van de onderzoeker aan de afdeling.

- Bijkomende supportieve behandeling:
  - Bloeddrukbehandeling: streefwaarde < 130/80 mmHg. Bij voorkeur ACE-remmer, evt. ARB
  - Bij hypercholesterolaemie langer dan 6 maanden na start behandeling: toevoegen HMG-CoA-reductase remmer. Streefwaarde LDL cholesterol < 2.6 mmol/l
  - Matige zoutbeperking
  - Overwegen orale antistolling indien serum albumine < 20 g/l of < 25 g/l met ernstige proteïnurie (>8 g/dg) en ernstig oedeem. Streefwaarde INR 2,0- 3,0

Screening, inclusie en follow-up:

Een gedetailleerd Follow-up schema is bijgevoegd als bijlage 2.

Patiënten worden voor reguliere patiëntzorg verwezen voor een "2 uurs- proteinuriemeting". Gegevens van deze meting worden gebruikt als screening. Tijdens deze screening wordt anamnese en lichamelijk onderzoek verricht. Daarnaast vindt een proteïnuriemeting volgens gestandaardiseerd protocol plaats (zie bijlage 1), om het risicoprofiel van patiënt te bepalen aan

de hand van de β2-microglobuline excretie. Bij gebleken geschiktheid wordt patiënt uitgebreid schriftelijk en mondeling geïnformeerd over de studie en wordt infromed consent gevraagd. Hierbij geldt een bedenktijd van minimaal 1 week.

Na het tekenen van de informed consent wordt de behandeling binnen 6 weken gestart.

Op het moment van inclusie en start van de behandeling (t=0) vindt bloed- en urineonderzoek plaats.

Follow-up tijdens de behandeling omvat maandelijks bezoek aan arts gedurende de eerste 3 maanden van de studie, daarna 2 maandelijks. Tijdens deze bezoeken wordt bloed- en urineonderzoek verricht, na 3 en 9 maanden wordt ook de gestandaardiseerde proteïnuriemeting herhaald.

Na het beëindigen van de behandeling (na 9 maanden) zijn er visites aan het eind van maand 12, 15, 18 en 24 gepland. Op medische indicatie vinden zo nodig frequentere visites plaats.

Stop behandeling:

De behandeling in het kader van het onderzoek zal direct worden gestopt indien:

1. De patiënt hierom verzoekt.
2. In het geval van onacceptabele bijwerkingen (een SSAR of SUSAR, als gedefinieerd in GCP richtlijnen), bij elke overgevoeligheidsreactie als beschreven in de productinformatie van Synacthen Depot (zie bijlage D2).
3. Bij stijging van het serum kreatinine met meer dan 25% ten opzichte van de waarde aan het begin van de studie of stijging van het serum kreatinine tot > 135 µmol/l. Op dat moment zal behandeling met cyclofosfamide en prednison worden gestart. Follow-up gegevens van deze patiënten zullen wel volgens het studieprotocol verzameld worden.

Follow-up gegevens van patiënten bij wie de behandeling is gestopt, zullen ook na stoppen van de behandeling volgens protocol verzameld worden, mits de patiënt hiermee instemt.

Patiënten bij wie de behandeling gestopt is, zullen vervolgens volgens de huidige standaard behandeld worden. Deze behandeling kan plaatsvinden in het UMC St Radboud of bij de eigen nefroloog in de regio, afhankelijk van de voorkeur van patiënt.

Patiënten bij wie de behandeling gestopt is, zullen niet vervangen worden in het onderzoek, d.w.z. er worden geen extra patiënten geïncludeerd.

Beoordeling van de werkzaamheid:

Nadat de behandeling bij de eerste 6 patiënten is beëindigd, zal een interimanalyse plaatsvinden. Inclusie van de tweede groep patiënten (n =14), zal slechts plaatsvinden indien de behandeling haalbaar en praktisch uitvoerbaar is gebleken (> 85% van de injecties op het juiste tijdstip toegediend).

Werkzaamheidparameters zijn de afname van de proteïnurie, het toenemen van het serum albumine en de verandering in het serum kreatinine. Deze parameters worden bij alle bezoeken bepaalt. Analyse van deze parameters vind plaats aan het eind van de behandeling na 9 maanden en na 24 maanden.

Stijging van het serum kreatinine met meer dan 25% of >135 μmol/l zal beschouwd worden als een gebrek aan werkzaamheid, waarna de behandeling gestopt zal worden (zie bovenstaande paragraaf).

Beoordeling van de veiligheid:

De studie medicatie zal worden uitgegeven en bewaard volgens de voorschriften van de fabrikant (zie bijlage A) en de afdeling Apotheek/ Klinische Farmacie volgens de richtlijnen van GCP en GMP. In dit geval wordt de studie medicatie opgeslagen en bewaard bij temperaturen tussen de 2 en 8 graden Celsius. Middels een “log” in de koelkast zal er gecontroleerd worden of niet van deze temperatuur is afgeweken.

Na elke injectie met de studiemedicatie worden patiënten 30 minuten geobserveerd in verband met het eventueel optreden van een overgevoeligheidsreactie.

Veiligheidsparameters in het bloed worden tijdens elk bezoek gecontroleerd (onder andere serum kalium, glucose, leverenzymen, serum kreatinine).

Tijdens elk bezoek wordt proefpersonen gevraagd naar eventuele bijwerkingen. Bijwerkingen worden genoteerd in zowel de status van de patiënt als in het Case Record Form (CRF). Daarnaast vindt een aparte registratie plaats van ongewenste voorvallen (AE’s) voor alle patiënten binnen het onderzoek middels specifieke formulieren (bijlage 3).

Ernstige ongewenste voorvallen (serious adverse events, SAE’s) worden binnen 24 uur door de onderzoeker gemeld aan de verrichter van het onderzoek, middels een speciaal formulier (bijlage 3).

De verrichter bepaald of er sprake is van een relatie van het SAE met de studie medicatie (bijwerking/ adverse reaction) en indien dit het geval is, of er sprake is van een verwachte danwel onverwachte bijwerking (SSAR versus SUSAR).

Een SUSAR zal binnen de wettelijk gestelde termijn worden gerapporteerd aan de METC, CBG en de bevoegde instantie. Rapportage van SSAR’s zal jaarlijks geschieden, conform de regelgeving.

De nazorg aan patiënten met een adverse event zal afhankelijk van de (klinische) hulpvraag geschieden binnen de reguliere patiëntenzorg.

Voortijdig afbreken van de studie: de studie zal voortijdig worden afgebroken indien zich meer dan 1 SUSAR voordoet bij de eerste groep van 6 patiënten of meer dan 3 SUSARs in de totale groep van 20 patiënten.

(Statistische) analyse:

Keuze van de groepsgrootte:
In eerste instantie is gekozen voor start van de studie met 6 patiënten. Dit beperkte aantal is ingegeven door het feit dat de haalbaarheid en praktische uitvoerbaarheid van behandeling met ACTH 2 keer per week nog niet getoetst is.

Indien de studie goed praktisch uitvoerbaar blijkt, zullen wij de studiegrootte uitbreiden naar 20 patiënten totaal. Klinisch uitgangspunt is hierbij dat in eerdere studies met ACTH 80- 90% van de patiënten een remissie bereikt.[28;30] Het ging in deze studies echter niet specifiek om hoog risico patiënten. De kans dat een hoog risico patiënt een spontane remissie bereikt ligt rond de 10%.[25] Stellen we als uitgangspunt dat ACTH in 60% van de patiënten een remissie induceert, dan zouden in een gerandomiseerde studie met 2 armen, per groep 20 patiënten nodig zijn om een verschil aan te tonen met een power van 0.9 en een α van 0.05. Hoewel de huidige studie slechts 1 arm omvat zouden we met deze 20 patiënten een verschil met een theoretische controle groep kunnen aantonen.

Bij ontbreken van gegevens of bij patiënten bij wie de follow-up vroegtijdig is beëindigd, zullen de laatst bekende gegevens als eindgegevens worden betrokken in de analyse.

Bij analyse van de effectiviteit van ACTH (secundaire eindpunten) zullen de gegevens van alle patiënten die geïncludeerd zijn in de studie worden geanalyseerd, ook indien zij de behandeling voortijdig hebben moeten staken (intention to treat-analyse). In tweede instantie zal apart worden gekeken naar die patiënten die de behandeling volledig hebben afgerond (per protocol analyse).

Kwaliteitsbeheersing en -borging

Een onafhankelijke monitor is aangesteld ten behoeve van dit onderzoek.

- De monitor zal voor start van het onderzoek verifiëren of de faciliteiten, apparatuur en staf geschikt zijn om het onderzoek veilig en naar behoren uit te voeren.
- De monitor zal gedurende het onderzoek eens per 6 maanden en nadat de laatste patiënt de behandeling beëindigd heeft, een monitorprocedure uitvoeren, met speciale aandacht voor de informed consent verklaringen, rapportage van adverse events en de correctheid van de gegevens op het CRF in vergelijking met de brondocumenten.
- De monitor zal van zijn bezoeken een rapport opmaken.

Inzage in brongegevens/brondocumenten:

De onderzoekers zullen op verzoek direct inzage verschaffen in brongegevens/ brondocumenten ten behoeve van aan het onderzoek betrokken monitors, auditers, beoordeling van de METC of door de bevoegde autoriteiten.
Patiënten wordt voor deelname (schriftelijk) toestemming gevraagd voor directe inzage in zijn medisch dossier ten behoeve van het onderzoek door bovengenoemde personen/instanties.

Ethische aspecten

Patiënten die voor deze studie in aanmerking komen zijn mensen met een bewezen nierziekte (in casu MGN) en een hoog risico op nierfalen. Hoewel er in de literatuur geen consensus bestaat over de behandeling bij deze patiënten is het algemeen aanvaard dat deze patiënten enige vorm van behandeling behoeven.
Op dit moment is de standaardbehandeling in ons centrum een combinatietherapie van cyclofosfamide en prednison, waarbij de behandeling gestart wordt op het moment dat de nierfunctie achteruitgaat (stijging van het serum kreatinine). Aangezien deze standaard behandeling gepaard gaat met veel bijwerkingen wordt gezocht naar een alternatief.

In dit onderzoeksvoorstel wordt een alternatieve behandeling in de vorm van ACTH onderzocht. Deze behandeling heeft in een drietal studies van twee onafhankelijke studiegroepen tot veelbelovende resultaten geleid.[28-30] Met deze behandeling wordt begonnen op het moment dat de nierfunctie nog normaal is. Dit betekent dat er bij onvoldoende werkzaamheid nog voldoende tijd is om patiënten alsnog met de standaardtherapie te behandelen. Onwerkzaamheid, gedefinieerd als een stijging van het serum kreatinine met meer dan 25% onder behandeling, is een stopcriterium voor het onderzoek. Patiënten lopen door deelname aan de studie dus nooit vertraging op in het starten van de bekende standaardbehandeling volgens de huidige criteria.

De toediening van de studiemedicatie geschiedt middels intramusculaire injectie door een gekwalificeerd persoon in een medische setting. Na toediening van iedere injectie wordt een proefpersoon gedurende 30 minuten geobserveerd. De totale behandeling bestaat uit 59 injecties in een periode van 9 maanden. Dit leidt tot een aanzienlijke (tijds-)belasting voor de proefpersoon. Indien echter, zoals in eerdere studies aangetoond, behandeling met ACTH leidt tot een remissie van de proteïnurie, wordt behandeling met cyclofosfamide vermeden.
Voor de patiëntengroep waarop deze studie gericht is, lijkt de belasting daarom een zeer redelijke prijs voor het vermijden van de behandeling met cyclofosfamide en de daarmee samenhangende bijwerkingen.
Hierbij moet wel worden toegegeven dat er nog onvoldoende bekend is over de lange termijn resultaten na behandeling met ACTH. Voor patiënten met een kinderwens kan echter alleen al het uitstellen van de behandeling met cyclofosfamide voordeel opleveren, zelfs als een later recidief alsnog behandeling met dit middel noodzakelijk maakt.

Verwerking en vastlegging van gegevens

Gegevens zullen worden vastgelegd in de volgende brondocumenten:
- medische status
- uitslagen gestandaardiseerde proteïnuriemeting in database "Proteïnurie"

Van daaruit zullen gegevens worden overgenomen in het voor de studie vervaardigde Case Record Form (zie bijlage 4). Een aantal gegevens zal direct worden opgenomen in dit CRF, te weten de checklist met betrekking tot de in- en exclusie criteria, de registratie van de toegdiende injecties Synacthen Depot en de checklist met betrekking tot de rapportage van eventuele bijwerkingen.

Het CRF is herleidbaar gecodeerd naar de patiënt.
Verdere verwerking van gegevens voor analyse zal plaatsvinden in een Accesdatabase, waarna de gegevens niet herleidbaar naar een SPSS database zullen worden getransporteerd. De gegevens worden anoniem en niet herleidbaar verwerkt in de uiteindelijk onderzoeksresultaten.

Financiering en verzekering

Financiering:

Dit onderzoek is investigator initiated en wordt gefinancierd door de afdeling Nierziekten.
Het betreft hier gelden vanuit de 1e geldstroom.

Patiënten die deelnemen aan het onderzoek ontvangen hiervoor geen vergoeding.

Er zijn geen conflicterende (financiële) belangen in deze studie.

Verzekering:

Conform de WMO is voor de proefpersonen die deelnemen aan dit onderzoek door het UMC St Radboud een verzekering afgesloten. Deze verzekering dekt de eventuele schade door letsel als gevolg van deelname aan het onderzoek, die zich openbaart gedurende deelname aan het onderzoek of binnen 5 jaar ná deelname aan het onderzoek. De verzekering dekt schade tot een maximumbedrag van Euro 453.780 (1 miljoen gulden) per proefpersoon (met dien verstande dat een maximumbedrag van Euro 6.806.703 (15 miljoen gulden) beschikbaar is voor het vergoeden van alle schade die door deelnemers aan het onderzoek wordt geleden en een maximumbedrag van Euro 9.075.604 (20 miljoen gulden) per verzekeringsjaar geldt voor alle onderzoeken die door het UMC St Radboud worden verricht.

Door de verzekering wordt niet de schade gedekt:
- die optreedt bij nakomelingen als gevolg van een nadelige inwerking van het onderzoek op het genetisch (= erfelijk) materiaal van patiënten;
- waarvan op grond van de aard van het onderzoek (nagenoeg) zeker was dat deze zich zou voordoen;
- die ook zou zijn opgetreden als de patiënt niet aan het onderzoek zou hebben deelgenomen.

Naam en adres van de verzekeraar:
Akkermans van Elten Assurantiën BV
Postbus 1275
6501 BG Nijmegen
Tel: 024 - 3511000
contactpersoon: de heer A.J.M. Swildens

Administratie en publicatiebeleid

De sponsor en onderzoeker zullen jaarlijks een voortgangsrapportage doen aan de METC. Dit zal gecombineerd worden met de melding van SSAR's. Er zal informatie worden gegeven over de datum van inclusie van de 1^{e} proefpersoon, het aantal patiënten geïncludeerd , het aantal patiënten dat de studie heeft gecompleteerd, SAE's en SSAR's, andere problemen en eventuele amendementen.

De verrichter zal de bevoegde METC binnen een periode van 90 dagen na het beëindigen van de studie informeren. Het einde van de studie is gedefinieerd als het laatste bezoek van de laatste patiënt.

Indien de studie vroegtijdig wordt beëindigd, zal de verrichter de METC en de bevoegde Instantie binnen 15 dagen op de hoogte stellen, inclusief de redden van het beëindigen van de studie.

Resultaten verkregen uit deze studie zullen worden gepubliceerd in abstract en/of postervorm, dan wel als artikel worden aangeboden aan gerenommeerde, peer-reviewed tijdschriften. In deze publicaties zullen geen gegevens staan die direct herleidbaar zijn tot de deelnemende patiënten.

Reference List

1. Tiebosch ATMG, Wolters J, Frederik PM *et al*. Epidemiologie van primaire glomerulonefritis in de regio Zuid-Limburg. *Ned Tijdschr Geneeskd* 1986; 130: 357-360

2. du Buf-Vereijken PW, Branten AJW, Wetzels JFM. Idiopathic membranous nephropathy: outline and rationale of a treatment strategy. *Am J Kidney Dis* 2005; 46: 1012-1029

3. Ponticelli C, Zucchelli P, Passerini P *et al*. A 10-year follow-up of a randomized study with methylprednisolone and chlorambucil in membranous nephropathy. *Kidney Int* 1995; 45: 1600-1604

4. Lewis EJ. Idiopathic membranous nephropathy--to treat or not to treat? *New Engl J Med* 2008; 329: 85-89

5. Perna A, Schiepatti A, Zamora J, Giuliano GA, Braun N, Remuzzi G. Immunosuppressive treatment for idiopathic membranous nephropathy: a systematic review. *Am J Kidney Dis* 2004; 44: 401

6. Hogan SL, Muller KE, Jennette JC, Falk RJ. A review of therapeutic studies of idiopathic membranous glomerulopathy. *American Journal of Kidney Diseases* 1995; 25: 862-875

7. Cattran DC, Delmore T, Roscoe J *et al*. A randomized controlled trial of prednisone in patients with idiopathic membranous nephropathy. *New Engl J Med* 1989; 320: 210-215

8. Cameron JS, Healy MJR, Adu D. The Medical Research Council trial of short-term high-dose alternate day prednisolone in idiopathic membranous nephropathy with nephrotic syndrome in adults. The MRC Glomerulonephritis Working Party. *Q J Med* 1990; 74: 133-156

9. Branten AJW, Reichert LJM, Koene RAP, Wetzels JFM. Oral cyclophosphamide versus chlorambucil in the treatment of patients with membranous nephropathy and renal insufficiency. *Q J Med* 1998; 91: 359-366

10. Imperiale TF, Goldfarb S, Berns JS. Are cytotoxic agents beneficial in idiopathic membranous nephropathy? A meta-analysis of the controlled trials. *J Am Soc Nephrol* 1995; 5: 1543-1558

11. Cattran DC, Greenwood C, Ritchie S *et al*. A controlled trial of cyclosporine in patients with progressive membranous nephropathy. Canadian Glomerulonephritis Study Group. *Kidney International* 1995; 47: 1130-1135

12. Ponticelli C, Villa M. Does cyclosporin have a role in the treatment of membranous nephropathy? *Nephrol Dial Transpl* 1999; 14: 23-25

13. Goumenos DS, Kalliakmani P, Tsakas S, Sotsiou F, Vlachojanni JG. The remission of nephrotic syndrome with cyclosporin treatment does not attenuate the progression of idiopathic membranous nephropathy. *Clin Nephrol* 2004; 61: 17-24

14. Muirhead N. Management of idiopathic membranous nephropathy: evidence-based recommendations. *Kidney Int Suppl* 1999; 70: 55

15. Ahuja M, Goumenos DS, Shortland JR, Gerakis A, Brown CB. Does immunosuppression with prednisolone and azathioprine alter the progression of idiopathic membranous nephropathy? *American Journal of Kidney Diseases* 1999; 34: 521-529

16. Goumenos DS, Ahuja M, Davlouros P, El Nahas AM, Brown CB. Prednisolone and azathioprine in membranous nephropathy: a 10-year follow-up study. *Clin Nephrol* 2006; 65: 317-323

17. Cattran DC. Mycophenolate mofetil and cyclosporine therapy in membranous nephropathy. *Semin Nephrol* 2003; 23: 272-277

18. Branten AJW, du Buf-Vereijken PWG, Vervloet M, Wetzels JFM. Mycophenolate mofetil in idiopathic membranous nephropathy: a clinical trial with comparison to a historic control group treated with cyclophosphamide. *American Journal of Kidney Diseases* 2007; 50: 248-256

19. Ruggenenti P, Chiurchiu C, Abbate M *et al*. Rituximab for idiopathic membranous nephropathy: who can benefit? *Clin J Am Soc Nephrol* 2008; 1: 738-748

20. Fervenza FC, Cosio FG, Erickson SB *et al*. Rituximab treatment of idiopathic membranous nephropathy. *Kidney International* 2008; 73: 117-125

21. Honkanen E, Tornroth T, Gronhagen-Riska C, Sankila R. Long-term survival in idiopathic membranous glomerulonephritis: can the course be clinically predicted? *Clin Nephrol* 1994; 44: 137-139

22. Cattran DC, Pei Y, Greenwood C, Ponticelli C, Passerini P, Honkanen E. Validation of a predictive model of idiopathic membranous nephropathy: its clinical and research implications. *Kidney Ínt* 1997; 51: 901-907

23. Reichert LJM, Koene RAP, Wetzels JFM. Urinary excretion of b2-microglobulin predicts renal outcome in patients with idiopathic membranous nephropathy. *J Am Soc Nephrol* 1995; 6: 1666-1669

24. Bazzi C, Petrini C, Rizza V *et al*. Urinary excretion of IgG and a1-microglobulin predicts clinical course better than extent of proteinuria in membranous nephropathy. *Am J Kidney Dis* 2001; 38: 240-248

25. Branten AJW, du Buf-Vereijken PW, Klasen IS *et al*. Urinary excretion of b2-microglobulin and IgG predict prognosis in idiopathic membranous nephropathy: a validation study. *J Am Soc Nephrol* 2005; 16: 169-174

26. du Buf-Vereijken PW, Branten AJ, Wetzels JF. Cytotoxic therapy for membranous nephropathy and renal insufficiency: improved renal survival but high relapse rate. *Nephrol Dial Transplant* 2004; 19: 1142-148

27. Faurschou M, Sorensen IJ, Mellemkjaer L *et al*. Malignancies in Wegener's granulomatosis: incidence and relation to cyclophosphamide therapy in a cohort of 293 patients. *The Journal of Rheumatology* 2007; 35: 100-105

28. Berg AL, Nillson-Ehle P, Arnadottir M. Beneficial effects of ACTH on the serum lipoprotein profile and glomerular function in patients with membranous nephropathy. *Kidney International* 1999; 56: 1534-1543

29. Berg AL, Arnadottir M. ACTH-induced improvement in the nephrotic syndrome in patients with a variety of diagnoses. *Nephrol Dial Transpl* 2004; 19: 1305-1307

30. Ponticelli C, Passerini P, Salvadori M *et al*. A randomized pilot trial comparing methylprednisolone plus a cytotoxic agent versus synthetic adrenocorticotropic hormone

in idiopathic membranous nephropathy. *American Journal of Kidney Diseases* 2006; 47: 233-240

31. Berg AL, Rafnsson AT, Johannsson M, Dallongeville J, Arnadottir M. The effects of adrenocorticotrophic hormone and an equivalent dose of cortisol on the serum concentrations of lipids, lipoproteins, and apolipoproteins. *Metabolism* 2006; 55: 1083-1087

32. Keane WF. Lipids and the kidney. *Kidney International* 1994; 46: 910-920

33. Arnadottir M. Treatment scheme ACTH. 2008.
Ref Type: Personal Communication

34. European Medicines Agency. Guidance on Good Clinical Practice (CPMP/ICH/135/95). www.emea.eu/pdfs/human/ich/013595en.pdf . 2006.
Ref Type: Electronic Citation

Protocol samenvatting

Titel: Synthetisch adrenocorticotroop hormoon (ACTH) bij de behandeling van patiënten met een membraneuze glomerulonefritis (MGN) en een hoog risico op nierfalen. Een pilot-studie.

Korte titel: ACTH in hoog-risico patiënten met MGN.

Eudra-CT nummer: 2008-001647-19

Klinische fase: II

Verrichter: Universitair Medisch Centrum St Radboud

Studie opzet: Open, niet-gecontroleerde pilotstudie.

Doel:

Primair doel:

- Evalueren van de haalbaarheid en praktische uitvoerbaarheid van therapie met synthetisch ACTH bij MGN.

Secundaire doelen:

- Evalueren van de effectiviteit van ACTH bij MGN, uitgedrukt in het percentage bereikte remissies.
- Evalueren van de effectiviteit van ACTH bij MGN, uitgedrukt in de verandering van de nierfunctie (kreatinine klaring volgens MDRD) in de tijd en het aantal recidieven.
- Evalueren van de veiligheid van ACTH bij MGN, uitgedrukt in het aantal bijwerkingen.
- Inzicht verkrijgen in het werkingsmechanisme van ACTH bij MGN door nagaan van het effect van ACTH op de endogene cortisol/cortison productie.

Proefpersonen: Start met 6 patiënten voor uitvoerbaarheidsonderzoek. Indien praktische haalbaarheid bewezen: uitbreiding met 14 patiënten tot 20 totaal.

Inclusie criteria:

- Leeftijd 18-75 jaar
- Idiopatische membraneuze glomerulonefritis, biopsie bewezen.
- Nefrotisch syndroom: proteïnurie ≥ 3.5 g/dag en serum albumine ≤ 30 g/l
- Normale of licht gestoorde nierfunctie (eGFR > 60 ml/min/1.73m^2)
- Hoog risico op nierfalen: β2-microglobuline excretie > 500 ng/min
- Relatieve contra-indicatie voor behandeling met cyclofosfamide:
  - Kinderwens
  - Hoge leeftijd (> 60jaar)
  - eerdere behandeling met cyclofosfamide
  - intolerantie voor cyclofosfamide

Exclusie criteria:

- Klinische, biochemische of histologische tekenen van een onderliggende systeemziekte.
- Infectieziekte (inclusief latente tuberculose en/of latente amoebiasis)
- Actief ulcuslijden (maag/duodenum)
- Zwangerschap, borstvoeding, inadequate anticonceptie
- Klinische tekenen van niervenetrombose
- Astma en/ of bekende hypersensitiviteitsreacties
- Allergische reactie op synthetisch ACTH in het verleden

Behandeling:

1. Synacthen depot (Novartis, Basel, Zwitserland) 1mg/ml voor intramusculaire injectie. Behandeling gedurende 9 maanden, volgens onderstaand schema.

Dosering opbouwen van 1x 1mg week naar 2x 1 mg per week in een periode van 8 weken.
Daarna behandeling met 2 x 1 mg per week gedurende 18 weken.
Hierna geleidelijke afbouw van de medicatie gedurende 13 weken.

Totaal aantal injecties per patiënt: 59
De injecties worden intramusculair toegediend door een verpleegkundige of arts. Na de injectie wordt de patiënt gedurende 30 minuten geobserveerd vanwege mogelijke overgevoeligheidsreacties.

2. Bijkomende supportieve behandeling:

- Bloeddrukbehandeling: streefwaarde < 130/80 mmHg. Bij voorkeur ACE-remmer, evt. ARB
- Bij hypercholesterolaemie langer dan 6 maanden na start behandeling: toevoegen HMG-CoA-reductase remmer. Streefwaarde LDL cholesterol < 2.6 mmol/l
- Matige zoutbeperking
- Overwegen orale antistolling indien serum albumine < 20 g/l of < 25 g/l met ernstige proteïnurie (>8 g/dg) en ernstig oedeem. Streefwaarde INR 2,0- 3,0

Stop behandeling:

1. Op verzoek van de patiënt of behandelend arts
2. In het geval van onacceptabele bijwerkingen
3. Bij stijging van het serum kreatinine met meer dan 25% ten opzichte van de waarde aan het begin van de studie of stijging van het serum kreatinine tot > 135 µmol/l. Op dat moment zal behandeling met cyclofosfamide en prednison worden gestart. Follow-up gegevens van deze patiënten zullen wel volgens het studieprotocol verzameld worden.

Eindpunten:

Primair

- Haalbaarheid van 2 keer per week toedienen van een intramusculaire injectie synthetisch ACTH gedurende 9 maanden (% injecties op juiste tijdstip toegediend).

Secundair

- Aantal patiënten dat de behandeling gedurende 9 maanden completeert.
- Patiëntbelasting van de behandeling (subjectieve belasting op schaal 1 tot 10) .
- Aantal remissies, zowel compleet als partieel (proteïnurie resp. < 0.2 g/dg of <2.0 g/dg)
- Aantal complete remissies (proteïnurie < 0.2 g/dg)
- Aantal partiële remissies (proteïnurie <2.0 g/dg)
- Verandering van de proteïnurie (indien geen remissie bereikt)
- Verandering van nierfunctie (veranderde klaring)
- Bijwerkingen

1 Assesments: Algemeen

- Anamnese en lichamelijk onderzoek tijdens screening voor inclusie.
- Injectie van studie medicatie volgens schema via de polikliniek of via regionaal ziekenhuis (dialyseafdeling).
- Maandelijks bezoek aan arts gedurende de eerste 4 maanden van de studie, daarna 2 maandelijks. Na het beëindigen van de behandeling (na 9 maanden) visites aan het eind van maand 12, 15, 18 en 24.

Laboratorium tests

- Biochemische en hematologische evaluatie tijdens screening en tijdens elk bezoek.
- Urine analyse tijdens screening en tijdens elk bezoek
- Aan de start van de studie en na 3 en 9 maanden wordt een gestandaardiseerde urine meting verricht.
- Aan de start van de studie en na 3 en 9 maanden wordt bloed afgenomen voor bepaling van cortisol

Veiligheid/bijwerkingen

- Na elke injectie worden patiënten 30 minuten geobserveerd in verband met het eventueel optreden van een overgevoeligheidsreactie.
- Tijdens elk bezoek wordt proefpersonen gevraagd naar eventuele bijwerkingen.
- Veiligheidsparameters in het bloed worden tijdens elk bezoek gecontroleerd (onder andere serum kalium, leverenzymen, glucose).

BIJLAGE 1: PROCEDURE PROTEINURIEMETING

In ons ziekenhuis worden patiënten met proteïnurie geëvalueerd met behulp van een standaard protocol. Bij al deze patiënten worden gestandaardiseerde urine- en bloedmetingen verricht als hieronder beschreven.

De 2 dagen voorafgaand aan de meting is door de patiënten thuis reeds 24-uurs urine verzameld voor meting van de dagelijkse excretie van kreatinine en totaal eiwit.

Op de dag van de meting komen patiënten ’s ochtends nuchter naar de afdeling. Op de ochtend van de meting mogen geen diuretica gebruikt worden. Patiënten zijn geïnstrueerd om de avond voor hun komst naar het ziekenhuis 4000 mg Natriumbicarbonaat via tabletten in te nemen. Dit om te verzekeren dat de urine pH hoger is dan 6.0, hetgeen vereist is voor de meting van het eiwit β-2-microglobuline in de urine.
De meting wordt begeleid door een getrainde onderzoeksverpleegkundige. Na aankomst krijgen patiënten 375 tot 500 ml kraanwater te drinken, om diurese te forceren. Voor start van de meting wordt “uitgeplast”, waarna de werkelijke meting begint. Patiënten blijven gedurende 1 uur in horizontale positie liggen. Middels een automatische bloeddrukmeter wordt de bloeddruk gemeten, waarbij 10 opeenvolgende metingen met een interval van 5 minuten worden verricht en geregistreerd (Welch Allyn, Beaverton OR, USA). Na 1 uur wordt opnieuw uitgeplast, aldus wordt een getimed urine monster verkregen. Vervolgens wordt bloed afgenomen. Dit is het einde van de feitelijke metingprocedure. Na de meting volgt een kort bezoek aan de arts, waarbij de klachten van patiënt worden uitgevraagd en op indicatie een kort lichamelijk onderzoek wordt verricht.

In het bloedmonster worden de volgende parameters met standaard automatische technieken bepaald: natrium, kalium, calcium, fosfaat, ureum, kreatinine, cholesterol,triglycerdiden, HDL-cholesterol, LDL-cholesterol (berekend), Hb, hematocriet , ijzer en ferritine.
De concentraties van serum albumine, transferrine en IgG worden gemeten met immunonephelometrie met behulp van een BNII nephelometer (Behring, Marburg, Duitsland). Serum β-2-microglobuline wordt gemeten middels ELISA [coating, code A072, detection (HRP-conjugated); code P174, beide Dakopatts, Denmark].

In het urinemonster worden totaal eiwit en kreatinine bepaald met standaard automatische technieken. De concentraties van urine albumine, transferrine, α-1-microglobuline en IgG worden gemeten met immunonephelometrie en urine β-2-microglobuline wordt gemeten middels ELISA (beide volgens bovenstaande technieken).

BIJLAGE 2: FOLLOW-UP SCHEMA

Bezoek 1: screening voor mogelijke inclusie/ standaardbezoek proteïnuriemeting
- anamnese
- algemeen intern lichamelijk onderzoek
- proteïnuriemeting (als beschreven in bijlage B)
- naast de parameters in het bloed als bepaald bij de standaard proteïnuriemeting wordt aanvullend ASA,ALA, LDH, ACTH, cortisol en glucose bepaald.
- in de 24hrs urine als verzameld voor de standaard proteïnuriemeting wordt aanvullend cortisol bepaald

Bezoek 2 op t=0: start studie medicatie
- Check: informed consent geteken?
- anamnese
- meting bloeddruk, gewicht, beoordeling perifeer oedeem
- bloedafname (venapunctie)^
- verzamelen urineportie+

Bezoek 3 op t= 1 maand
- anamnese (met specifieke aandacht voor eventuele bijwerkingen)
- meting bloeddruk, gewicht, beoordeling perifeer oedeem
- nuchter bloedafname (venapunctie) *
- verzamelen 24hrs urine voor bepaling kreatinine, eiwit en cortisol

Bezoek 4 op t= 2 maanden
- anamnese (met specifieke aandacht voor eventuele bijwerkingen)
- meting bloeddruk, gewicht, beoordeling perifeer oedeem
- nuchter bloedafname (venapunctie) *
- verzamelen 24hrs urine voor bepaling kreatinine, eiwit en cortisol

Bezoek 5 op t= 3 maanden
- anamnese (met specifieke aandacht voor eventuele bijwerkingen)
- algemeen intern lichamelijk onderzoek (inclusief bloeddruk, gewicht, oedeem)
- proteïnuriemeting (als beschreven in bijlage B)
- naast de parameters in het bloed als bepaald bij de standaard proteinuriemeting wordt aanvullend ASA,ALA, LDH, ACTH, cortisol en glucose bepaald.
- in de 24hrs urine als verzameld voor de standaard proteïnuriemeting wordt aanvullend cortisol bepaald

Bezoek 6 op t= 5 maanden
- anamnese (met specifieke aandacht voor eventuele bijwerkingen)
- meting bloeddruk, gewicht, beoordeling perifeer oedeem
- nuchter bloedafname (venapunctie) *
- verzamelen urineportie+

Bezoek 7 op t= 7 maanden
- anamnese (met specifieke aandacht voor eventuele bijwerkingen)
- meting bloeddruk, gewicht, beoordeling perifeer oedeem
- nuchter bloedafname (venapunctie) *
- verzamelen urineportie+

Bezoek 8 op t= 9 maanden: eind van behandeling

- anamnese (met specifieke aandacht voor eventuele bijwerkingen)
- algemeen intern lichamelijk onderzoek (inclusief bloeddruk, gewicht, oedeem)
- proteïnuriemeting (als beschreven in bijlage B)
- naast de parameters in het bloed als bepaald bij de standaard proteinuriemeting wordt aanvullend ASA, ALA, LDH , ACTH, cortisol en glucose bepaald.
- in de 24hrs urine als verzameld voor de standaard proteïnuriemeting wordt aanvullend cortisol bepaald

Bezoek 9 op t= 12 maanden

- anamnese
- meting bloeddruk, gewicht, beoordeling perifeer oedeem
- nuchter bloedafname (venapunctie) [*]
- verzamelen urineportie[+]

Bezoek 10 op t= 15 maanden

- anamnese
- meting bloeddruk, gewicht, beoordeling perifeer oedeem
- bloedafname (venapunctie)[^]
- verzamelen urineportie[+]

Bezoek 11 op t= 18 maanden

- anamnese
- meting bloeddruk, gewicht, beoordeling perifeer oedeem
- bloedafname (venapunctie) [^]
- verzamelen urineportie[+]

Bezoek 12 op t= 24 maanden: einde van follow-up

- anamnese
- meting bloeddruk, gewicht
- bloedafname (venapunctie) [^]
- verzamelen 24hrs urine voor meting van totaal eiwit en kreatinine

Totaal aantal bezoeken: 12, waarvan 3x gedurende 2 uur voor proteïnuriemeting
Totaal aantal venapuncties: 12

* bij venapunctie tijdens maand 1 tot en met 12 wordt bloed afgenomen bij een nuchtere patient om de volgende parameters in het bloed te laten bepalen: natrium, kalium, ureum, kreatinine, albumine, hemoglobine, leukocyten, trombocyten, glucose, ASA, ALA, LDH, totaal cholesterol, HDL cholesterol, triglyceriden, LDL cholesterol (berekend), cortisol en ACTH

^ bij venapunctie tijdens t=0 en tijdens maand 15 t/m 24 wordt bloed afgenomen om de volgende parameters in het bloed te laten bepalen (patiënt hoeft niet nuchter te zijn): natrium, kalium, ureum, kreatinine, albumine, hemoglobine, glucose, totaal cholesterol

+ in de verzamelde urine porties wordt de volgende bepaling verricht: eiwit/kreatinine ratio

BIJLAGE 3: RAPPORTAGE EN BEOORDELING ADVERSE EVENTS EN SERIOUS ADVERSE EVENT (SAE)

Tijdens elk bezoek wordt proefpersonen gevraagd naar eventuele bijwerkingen. Bijwerkingen worden genoteerd in zowel de status van de patiënt als in het Case Record Form (CRF).

Voor classificering van bijwerkingen wordt gebruikt gemaakt van de definities volgens het GCP- richtsnoer, waarin gesproken wordt van ongewenste voorvallen (adverse events).
Aparte registratie plaats van ongewenste voorvallen (AE's) voor alle patiënten binnen het onderzoek vindt plaats middels onderstaande formulieren.

Ernstige ongewenste voorvallen (serious adverse events, SAE's) worden binnen 24 uur door de onderzoeker gemeld aan de verrichter van het onderzoek.

De verrichter bepaald of er sprake is van een relatie van het SAE met de studie medicatie (bijwerking/ adverse reaction) en indien dit het geval is, of er sprake is van een verwachte dan wel onverwachte bijwerking (SSAR versus SUSAR).

Een SUSAR zal binnen de wettelijk gestelde termijn van 15 dagen (7 dagen in het geval van een levensbedreigende of dodelijk voorval) worden gerapporteerd aan de METC, CBG (cq. EudraVigilance) en de bevoegde instantie (CCMO).

Rapportage van SSAR's zal jaarlijks geschieden aan de METC en de CCMO.

De nazorg aan patiënten met een adverse event zal afhankelijk van de (klinische) hulpvraag geschieden binnen de reguliere patiëntenzorg.

De acties die worden ondernomen naar aanleiding van een (S)AE worden in de status en in het CRF van patiënten genoteerd.

AE Registration Form for protocol "Treatment with synthetic adrenocorticotropic hormone(ACTH) in patients with membranous nephropathy and high risk for renal failure. A pilot study."

Subjectnr:	________	Initials:	__________
Event:	AE	Initial report/ Follow-up report	
Event nr.:	________		

**AE_Name**	Name of Adverse Event	________________________<br>________________________<br>________________________
**AE_Number**	Number of AE	___
**AE_Start**	Start date and time of AE	__________________
**AE_Stop**	Stop date and time of AE	___________________
**AE_Ongoing**	Ongoing Adverse Event	0 (No)<br>1 (Yes)<br>! (Missing)
**AE_Sympt**	Symptoms of Adverse Event	________________________<br>________________________<br>________________________<br>________________________
**AE_Action**	Action taken on AE	1 (None)<br>2 (Dose adjustment)<br>3 (Temporary stop)<br>4 (Permanent stop)<br>! (Missing)

Subjectnr:	**Initials:**	**Event:** AE	**Event nr.**

AE_Action Action taken on Adverse Event
1 (none)
2 (dose adjustment)
3 (temporary stop)
4 (permanent stop)
! (Missing)

AEAddTreat Additional treatment of AE
1 (no)
2 (medication: complete CRF)
3 (other: describe in note)
! (Missing)

AE_AddTreat1 If other treatment, specify
____

____

____

AERelation Relation of AE to study medication?
1 (not related)
2 (unlikely related)
3 (possibly related)
4 (probably related)
5 (definitely related)
! (Missing)

AE_Intensity Intensity of AE according to NCI CTC _____

AE_Severity Severity of Adverse Event
1 (mild)
2 (moderate)
3 (severe)

AE-SAE Is this a Serious AE
0 No
1 Yes

If Yes, reason:
1 (Prolonged) hospitalization
2 Life-threatening
3 Persistent or significant disability or incapicity
4 Congenital anomaly or birth defect
5 Death

If SAE complete SAE form and report!

Reported on: ________________________ (date)

By: _________________________ (name investigator)

_________________________ (signature)

SAE Registration Form for protocol "Treatment with synthetic adrenocorticotropic hormone(ACTH) in patients with membranous nephropathy and high risk for renal failure. A pilot study."

Subjectnr:	________	Initials: __________
Event:	SAE	Initial report/ Follow-up report
Event nr.:	________	

**SAE**	Has any SAE taken place?	0 (No)<br>1 (Yes)<br>! (Missing)
**SAE_Name**	Name of Serious Adverse Event	_______________________<br>________________________<br>________________________
**SAE_Number**	Number of SAE	___
**AE_Number**	Number of Adverse Event	___
**SAE_Action**	Action taken on SAE	1 (None)<br>2 (Dose adjustment)<br>3 (Temporary stop)<br>4 (Permanent stop)<br>! (Missing)
**SAE_AddTreat**	Additional treatment on SAE	1 (No)<br>2 (Med: compl ConMedForm)<br>3 (Other: describe in note)<br>! (Missing)
**SAE_AddTreat1**	If other treatment, specify	

____

____

____

____

**SAE_Relation**	Relation of SAE to studymedication?	1 (Not likely)<br>2 (Unlikely related)<br>3 (Possibly related)<br>4 (Probably related)<br>5 (Definitely related)<br>! (Missing)

**Subjectnr:**	**Initials:**	**Event:** SAE	**Event nr.**

**SAE_Start**	Start date and time of SAE	___________________
**SAE_Stop**	Stop date and time of SAE	____________________
**SAE_Ongoing**	Ongoing Serious Adverse Event	0 (No)<br>1 (Yes)<br>! (Missing)
**SAE_ActStart**	Start action taken on SAE (date)	___________________
**SAE_ActStop**	Stop action taken on SAE	____________________
**SAE_Expected**	Could the SAE be expected? If SAE might be related and is unexpected, complete SUSAR form and report immediately according to SOP.	0 (No)<br>1 (Yes)<br>! (Missing)

Reported on: ________________________ (date)

By: _________________________ (name investigator)

__________________________ (signature)

Received and processed on: ________________________ (date)

By: _________________________ (name sponsor)

__________________________ (signature)

In case of SUSAR:
Reported to:

METC ____________ (date)

CCMO ____________ (date)

CBG _____________ (date)

EudraVigilance _____________ (date)
