# Supplementary material for: Synthetic ACTH in High Risk Patients with Idiopathic Membranous Nephropathy: A Prospective, Open Label Cohort Study
Source: PLoS One. 2015 Nov 12;10(11):e0142033. doi: 10.1371/journal.pone.0142033 (PMC4642982; doi:10.1371/journal.pone.0142033)
Supplement: S2 Protocol — (DOCX) [file pone.0142033.s002.docx]

**Protocol:**

**Treatment with synthetic adrenocorticotropic hormone (ACTH) in patients with membranous nephropathy and high risk for renal failure. A pilot study**

Version 1 dd. March 14th 2008

**Study coordinators:**

Drs.J.M.Hofstra

Prof.Dr.J.F.M.Wetzels

Dept of Nephrology 464

UMC St Radboud

PO box 9101

6500 HB Nijmegen, the Netherlands

T: +31 24 3614761

F: +31 24 3540022

**Content**

General information …...…………………………………………………………….. 2

Background ……………………………………………………….……………… 3

Aims ..………...………………………………………………………………….. 6

Study design …………………………………………………………………………. 7

Endpoints……………………………………………………………………. 7

Patients……………………………………………………………………… . 7

Treatment scheme ………………………………………………………… 8

Follow-up ……………………………………………………………………… 8

Withdrawal criteria ………..………………………………………………. 9

Efficacy parameters……. ……………………………………………….. 9

Safety parameters….. …………………………………………………….. 10

Statistical analyses …………………………………………………………… 11

Quality control …………. ……………………………………………………….. 12

Availability of source data/source documents ……………………………… 12

Ethical aspects ………………………………………………………………………. 13

Data collection and handling……. ………………………………………………. 14

Financial aspects and insurance ……………………………………………………….. 15

Administration and publication policy …………………………………………………. 16

References……………………………………………………………………….……… 17

Protocol summary …………………………………………………………… 20

**Appendices within protocol**

**1:** procedure standardized urine measurements ………..……………………………………..23

**2:** follow-up scheme ……………………………………………………………………….….21

**3:** Adverse Event (AE) and Serious Adverse Event (SAE) registration forms----……….…...25

**4:** Case Record Form ………………………………………..…………………….suppl.pages 1-24

**Appendices outside protocol (as needed for Ethical Approval Procedure):**

**D2:**  product information of the study agent ( incl. SPC’s and detailed treatment scheme)

**D3:** labels study medication

**E1:** patient information

**E2:** informed consent form

**General information:**

**Title:** Treatment with synthetic adrenocorticotropic hormone (ACTH) in patients with membranous nephropathy and high risk for renal failure. A pilot study.

**EudraCT number:** 2008-001647-19

**Protocol number**: 2008.1

**Protocol date:** version 3-14-2008

**Sponsor:** University Medical Centrum St Radboud

Prof.Dr.J.F.M.Wetzels, nephrologist

Dept of Nephrology 464

PO box 9101

6500 HB Nijmegen, the Netherlands

+31 24 3614761

**Monitor:** M.van Helden

Trial nurse

Dept of Nephrology 464

University Medical Centrum St Radboud

**Researcher:** Drs.J.M.Hofstra, resident internal medicine

**Trial site:**  Dept of Nephrology 464

UMC St Radboud

Geert Grooteplein 8

6525 GA Nijmegen

024 3614761

**Independent physician:** Dr.G.Vervoort, internist-nefroloog

Dept. 0f Internal Medicine and Dept. of Nephrology.

UMC St Radboud

**Other departments involved in this trial:**

Farmacy/ Dept. of Clinical Pharmacology 864

UMC St Radboud

Geert Grooteplein 8

6525 GA Nijmegen

024 3614761

Dept. of Laboratory Medicine 441

UMC St Radboud

Geert Grooteplein 8

6525 GA Nijmegen

024 3614761

**Background:**

Idiopathic membranous nephropathy (iMN) is the most common cause of nephrotic syndrome in adults, with an incidence in the Netherlands of 10 / million / year.^1^ In literature variable data are reported on the natural course of the disease. This is partly explained by the heterogeneity of the patient populations studied, including differences in age and sexe, renal function, degree of proteinuria and the duration of follow-up. The results of long term studies (with a follow-up of 9-14 years) can be broadly summarized as follows: without treatment, 14-56% of patients develop a spontaneous remission, while 34-62% progresses to renal failure. ^2;3^ After 10 years of follow-up persistent nephrotic syndrome was observed in only 4-9% of patients.

There is no consensus about the optimal treatment of patients with iMN.^4; 5^ The results of treatment with corticosteroids alone are disapponiting.^6-8^ Treatment with chlorambucil or cyclophosphamide combined with corticosteroids does show favorable effects with faster and more frequent remission of nephrotic syndrome and a better preservation of renal function.^3; 9; 10^ In our patient populaiton treatment with a combination of cyclophosphamide and prednisone proved to be more effective than the combination of chlorambucil and prednisone. In particular, during treatment with cyclophosphamide remission of the proteinuria occured more frequently (15/17 vs 5/15 patients), and a longer-lasting improvement of the renal function was seen with less adverse events.^9^
Alternative treatment strategies are not extensively studied. Treatment with cyclosporine appears less effective with frequent recurrence of proteinuria after withdrawal of therapy.^11-13^ Also, due to its nephrotoxicity cyclosporine is only limited applicable in patients with impaired renal function.^12^ Azathioprine is not effective, and treatment is frequently associated with adverse events.^14-16^ Mycophenolate mofetil (MMF) is not more effective in inducing remission than the alkylating agents and in our patient population, a high number of relapses was observed within 2 years of follow-up.^17; 18^ In recent studies, anti-B cell therapy (rituximab) appeared to be effective in some of the patients, while there was no response in others.^19; 20^

Given the natural course of the disease, with a spontaneous remission of proteinuria occuring in 50% of patients, and given the toxic effects of immunosuppressive therapy, there appear to be insufficient grounds to treat all patients with iMN with cytotoxic therapy. For this reason, there has been extensively searched for factors that predict the occurrence of renal insufficiency, with the intention to identify patients with a poor prognosis, and only to treat these patients.

In the past the predictive value of the degree and duration of proteinuria was underlined.^21: 22^ For the past 15 years we have prospectively followed patients with iMN and normal renal function. We observed that the urinary excretion of IgG and beta-2-microglobulin could predict the risk of renal dysfunction with a high sensitivity and specificity.^23^ The thresholds for excretion of beta-2-microglobulin and IgG were set at 500 ng / min and 250 mg / day. These findings were confirmed by Bazzi et al, who used α1 microglobulin (instead of beta 2 microglobulin) and IgG as predictive markers.^24^ In a recent study we have validated the predictive value of beta-2-microglobulin and IgG using the previous set threshold values. The combination of these markers predicts future renal function deterioration with a sensitivity and specificity of respectively 83% and 97% .^25^ Of the two markers, β2-microglobulin is the most important predictor of renal dysfunction.

It is generally accepted that patients with iMN with impaired renal function (serum creatinine> 135 μmol / l) should be treated, given the expected progression to ESRD. Based on the results of our validated predictive markers, we currently classify patients with iMN, nephrotic syndrome and normal renal function as either high or low risk patients, using the ß2-microglobulin excretion in the urine. Patients with a high risk of renal function loss are treated as soon as the serum creatinine start to increase, and treatment consist of cyclophosphamide and prednisone for 12 months. In patients of fertile age, cyclophosphamide will be replaced by azathioprine after 3 months of treatment.

Previously, we published long-term results of this treatment strategy.^26^ Because of the toxicity of the agent, treatment with cyclophosphamide has several drawbacks. Side effects, such as (pan) cytopenia occur frequently, necessitating dose reduction. In addition, there is an increased risk of serious infectious complications and long-term treatment is associated with an increased risk of (bladder) malignancies.^27^ Last but not least, cyclophosphamide treatment can lead to infertility. The risk of infertility increases with the cumulative dose, leading to the decision to switch therapy to the less effective azathioprine after 3 months of treatment, in dose patients who want to conceive in the future.

Due to the toxic effects of cyclophosphamide, the searc for an alternative treatment is ongoing. In a study assesing the effects of long-acting synthetic ACTH (adrenocorticotropic hormone) on the lipid profile of patients with nephrotic syndrome, Berg et al.observed an unexpected beneficial effect on the degree of the proteinuria in patients with iMN.^28^ During treatment with ACTH proteinuria decreased with 90%, and all five patients achieved remission. This effect was confirmed in a second study.^29^ In a recent randomized pilot study by Ponticelli et al., synthetic ACTH was as effective as treatment with steroids and an alkylating agent ( either cyclophosphamide or chlorambucil) in patients with iMN and nephrotic syndrome.^30^ In two groups of 16 patients the remission rate was equal (resp. 87% and 75%, difference in favor of ACTH is not significant). Of note, patients in this study had not been selected to be at high risk for future renal function deterioration. The side effects of synthetic ACTH in these studies were mild and reversible, and related to mineralo- and glucocorticoid effects. Side effects included glucose intolerance, mild Cushingoid appearance, fluid retention, insomnia, hyperpigmentation of the skin, and diarrhea. Hypersensitivity reactions to synthetic ACTH were not seen in these studies.

The exact mechanisms behind the effects of ACTH on proteinuria and glomerular function are not clear. ACTH is a pituitary hormone that stimulates the adrenal glands, , to the production of, in particular, glucocorticoids (cortisol). However, it is unlikely that the anti-proteinuric effect of synthetic ACTH completely relies on the increase in endogenous cortisol production. The dose of synthetic ACTH used in the studies of Berg et al., and Ponticelli, et al. (1mg twice a week) corresponds to about 300 mg of additional cortisol weekly, which is roughly the equivalent of 10-15 mg of prednisone daily.^31^ In previous studies, corticosteroid monotherapy in much higher dosages prooved not effective in patients with iMN.^5-7^

During the ACTH-induced cortisol synthesis, a large amount of endogenous cholesterol is consumed, which explains the beneficial effect of ACTH on the lipid profile. Although the hyperlipidemia accompanying nephrotic syndrome, might possibly be linked to the prognosis of kidney disease, it is not likely that the improvement in the lipid profile is the primary mechanism of action explaining the effect of synthetic ACTH.^32^ Improvement of the lipid profile is also achieved with the current treatment with HMG- reaches CoA reductase inhibitors. In addition to the effect on the cortisol synthesis ACTH also, to a lesser extent, stimulates the production of mineralocorticoid and androgenic steroids.

Despite the fact that the mechanism of action of ACTH in nephrotic syndrome is not yet unraveled, the treatment with this agents offers clear advantages. The use of high doses of steroids and cytotoxic cyclophosphamide - and thus the associated side effects - is avoided. Although there are no data on the long-term results or relapse rates after an ACTH-induced remission, even if treatment would only lead to a delay in treatment with alkylating agents, this could be an advantage for young patients who want to start a family in the near future. Possible disadvantage could be the practical feasibility of the treatment, since an intramuscular injection is to be administered to the patient twice a week. In the context of hypersensitivity reactions described by the manufacturer, the patient is to be observed by a care giver for 30 minutes after administration of an injection. Although this is a (time) burden for the patient, we believe that this outweighs the fact that the alkylating agents are avoided.

The aim of the present study is to assess whether a long-acting synthetic ACTH (tetracosactide hexaacetate / Synacthen Depot®) is a possible alternative to treatment with cyclophosphamide and prednisone in patients with iMN with normal renal function, but with a high risk of renal function loss. We want to include those patients with a relative contraindication to treatment with cyclophosphamide. These are young patients in the ferile age, patients over 60 years, in whom the risk of complications of treatment is increased, patients who have been treated in the past with cyclophosphamide or patients with intolerance to cyclophosphamide.

The current study is a pilot study: initially six patients will be treated with Synacthen Depot. The treatement regimen used, is equal to that used by the group of Berg and Árnadóttir, and was developed from the experience of their first 2 studies.^33^ In contrast to the schemes that were used in the studies published to date, in the current scheme, there is not only and incresing dose at start, but als a period of tapering at the end of treamtent. This was introduced to avoid the theoretical risk of relative adrenal insufficiency after prolonged treatment. After a period of 2 months with increasing dose, Synacthen Depot intramuscular injection is administered by a doctor or nurse / physician assistant for 4 months at the maximum dose of 1 mg twice a week. After 6 months, the dose is gradually tapered over a period of 3 months. (For the exact treatment schedule and detailed product description of Synacthen Depot see page 8 and Annex D2).
In these first six patients we will asses whether treatment with Synacthen Depot is practically and logistically feasible. If this is the case, an adiditional 14 patients will be treated according to the same protocol. Finally, the data of the total group of 20 patients will be analyzed with regard to the treatment results.

Note: Patients who show an increase in serum creatinine of more than 25%, or in whom the serum creatinine reaches a value of> 135 µmol/l during the study, are whithdrawn form the study medication and will be switch to standard therapy. Standard treatment includes treatment with cyclophosphamide and prednisone for 1 year; cyclophosphamide will replaced by azathioprine after 3 months in patients in fertile age.

The study will be conducted in accordance with the protocol, the rules of Good Clinical Practice and the applicable regulatory laws.^34^

**Aims:**

The aim of the present study is to evaluate whether ACTH is a possible alternative to treatment with cyclophosphamide and prednisone in patients with iMN with normal renal function, but with a high risk of renal function deterioration, and high risk of complications of the standard treatment with cyclophosphamide.

The reason for conducting the study is the fact that alternative treatment is warranted. The current treatment with cyclophosphamide and prednisone can lead to serious side effects. In addition, the treatment is not well applicable in young people, who want to start a family in the future, because of the risk of infertility associated with the use of cyclophosphamide.

**Primary aim:**

Evaluate the feasibility and practical logistic aspects of therapy with synthetic ACTH in six patients with iMN and a high risk of kidney failure.

The feasibility will be evaluated by scoring the percentage of injections that could be administered for the treatment at the correct time point.

**Secondary aims:**

- To assess the effectiveness of ACTH in the treatment of membranous nephropathy.

  If therapy with synthetic ACTH prooves feasible (> 85% of injections administered at the right time point), 14 additional patients will be enrolled in the study to assess the effectiveness (total 20 patients).
  If after treating the first six patients the study is considered not feasible, no additional patients will be enrolled and the analysis of the effectiveness will include only data of the first six patients.

  The effectiveness of the treatment is evaluated in terms of:
  - The percentage remissions achieved.
  - The change in renal function (estimated creatinine clearance according to MDRD) over time
  - The safety of ACTH expressed in terms of the number of side effects.
- To understand the mechanism of action of ACTH in membranous nephropathy by studying the effect of ACTH on endogenous cortisol / cortisone production.

**Study design**

This is an open label, uncontrolled pilot study.

**Endpoints:**

Primary

Feasibility of administration of an intramuscular injection of synthetic ACTH twice a week during 9 months; expressed in terms of the percentage of injections that is administered during the treatment at the correct time point. The correct time point is defined as the scheduled date ± 1 day.

Secondary

- The load of the treatment experienced by the patients after 9 months: shown on a scale of 1 to 10.

- Number of patients that complete the complete treatment period of 9 months.

- Number of remissions, both complete and partial (proteinuria <0.2 g / dy or <2.0 g / dy).

- Number of complete remissions (proteinuria <0.2 g / dy) at time points 9 and 24 months

- Number of partial remissions (proteinuria <2.0 g / dy) at time points 9 and 24 months

- Change in proteinuria (if not achieved remission) from t = 0 to 9 as well as 24 months, expressed as a percentage

- Changes in kidney function (altered eGFR from t = 0 to both 9 and 24 months, as a percentage

- Number of side effects.

**Patients:**

Initially 6 patients will be included. An additional number of 14 patients can be included if analysis of the primary endpoint (see below) has shown that there is a good feasibility of the treatment according to the study protocol.

Patients can be enrolled from the outpatient clinic of the department of Nephrology at UMC St. Radboud. In addition, nephrologists from the region, with whom there has been a long time partnership, will be asked to refer suitable patients for the study.

Inclusion criteria:

- Biopsy-proven idiopathic membranous nephropathy.
- Nephrotic syndrome: proteinuria > 3.5 g/day and serum albumine < 30 g/l
- Normal or mildly impaired renal function (eGFR > 60 ml/min, MDRD formula)
- High risk for renal failure: beta-2-microglobulin excretion > 500 ng/min
- Relative contra-indication for cyclophosphamide treatment:
  1. fertility and wish for (future) family expanding
  2. high age ( > 60 years)
  3. former cyclophosphamide treatment
  4. intolerance to cyclophosphamide

Exclusion criteria:

- Clinical,biochemical or histological signs of any underlying systemic disease
- Any infectious disease (including latent tuberculosis and/or latent amoebiasis)
- Active gastric or duodenal ulcers
- Pregnancy, lactation, inadequate contraceptives
- Clinical signs of renal vein thrombosis
- Asthma and /or any allergic conditions or hypersensitivity reactions
- Allergic reaction to synthetic ACTH in the past

**Treatment scheme:**

- Synthetic ACTH (tetracosactide hexaacetate/ Synacthen Depot) 1mg/ml for intramuscular injection. One injection of 1 ml contains 1 mg tetracosactide. Maximum dose 1 mg twice a week, total treatment period 9 months.
- At the start of the treatment, during a period of 2 months (8 weeks), the dose is increased of one injection per week to 2 injections per week . The injections thus should be administered on days 0, 7, 14, 21, 28, 32, 37, 42, 46, 51 and 56. Thereafter, the scheme continues for 4 months (18 weeks) with 2 injections per week. At month 7 tapering starts over a period of 3 months. The injections are then administered at day 186, 191, 196, 200, 205, 210, 217, 224, 231, 238, 245, 259 and 273. An injection may be administered up to 1 day prior to or after the scheduled day. Total number of injections per patient: 59 (see also Appendix D2)
- The injections are administered intramuscularly by a nurse or doctor. After the injection, the patient is observed for 30 minutes because of potential hypersensitivity reactions. The first injection will necessarily take place at UMC St Radboud.
- - Further injections may be adminsterd on the dialysis department at the regional hospital after personal instruction of the researcher in the department and after permission by the local ethical board.

Additional supportive treatment:

- Blood pressure: target <130/80 mmHg. Preferably ACE inhibitor, if necessary. ARB
- In case of hypercholesterolemia longer than 6 months after starting treatment: add HMG-CoA reductase inhibitor. Target LDL cholesterol <2.6 mmol / l
- Moderate salt restriction
- Consider oral anticoagulation if serum albumin <20 g / l or <25 g / l with severe proteinuria (> 8 g / dg), and severe edema. Target INR 2.0- 3.0

# Follow-up:

A detailed follow-up schedule is attached as Appendix 2.

Patients are referred to a "2 hour proteinuria measurement" for regular patient care. Data of this measurement are used as screening. Medical history is taken and physical examination is performed during this screening. In addition, there is a timed urine collection for proteinuria measurement according to a standardized protocol (see Appendix 1), in order to determine the risk profile of the patient based on urinary β2-microglobulin excretion. If eligible for study inclusion, the patient is informed extensively both on person and in writing about the study and informed consent is requested. Consent cna only be given after one week consideration time.

.

After signing the informed consent, the treatment is started within 6 weeks.

At the time of inclusion, and start of the treatment (t = 0), blood and urine tests will take place.

Follow-up during treatment includes monthly visits to the doctor during the first 3 months of the study, and visits every to months thereafter. During these visits blood and urine tests are performed , and after 3 and 9 months the standardized “proteinuria measurement” is repeated.

After the end of the treatment (after 9 months), visits are scheduled at the end of months12, 15, 18 and 24. If required on medical grounds, visits can be more frequent.

**Withdrawal criteria:**

The study treatment will be stopped immediately if:

1. on patients request.

2. In case of unacceptable side effects (a SSAR or SUSAR as defined by GCP guidelines), any hypersensitivity reaction as described in the SPC of Synacthen Depot (see Appendix D2).

3. serum creatinine increases more than 25% from baseline (start of study), or a increase in serum creatinine to> 135 mg / dl. In this case, treatment with cyclophosphamide and prednisone will be started. Follow-up data on these patients will be collected according to the study protocol.

Follow-up data from patients, in whom treatment is withdrawn, will be collected according to protocol after cessation of treatment, provided that the patient agrees with this.

Patients in whom treatment is withdrawn, will be treated according to the current standard. This treatment can take place in the Radboud UMC, or in regional hospitals, depending on the preference of the patient.

Patients in whom treatment is withdrawn, will not be replaced study.

**Efficacy parameters:**

After treatment is completed in the first six patients, an interim analysis will be performed. Inclusion of the second group of patients (n = 14), will proceed if the treatment has been shown to be feasible (> 85% of the injections administered at the right time point).

Efficacy parameters are the decrease in proteinuria, the increase in serum albumin and the change in serum creatinine. These parameters are determined at all visits. Analysis of these parameters will be performed at the end of the treatment after 9 months and after 24 months.

Increase in the serum creatinine of more than 25% or > 135 umol /l will be considered to be a lack of efficacy, after which the treatment will be stopped (see section above).

**Safety parameters:**

The study medication will be handled and stored according to the manufacturer's instructions (see Appendix A) at the Department of Pharmacy / Clinical Pharmacy following the guidelines of GCP and GMP. More specific, the study medication is stored at temperatures between 2 and 8 degrees Celsius. Through a "log" in the fridge it will be checked that there is no deviation from this temperature.

After each injection of study medication, patients are observed for 30 minutes because of the possible occurrence of a hypersensitivity reaction.

Safety parameters are checked during each visit in the blood (i.e. serum potassium, glucose, liver enzymes, serum creatinine).

During each visit, subjects are asked about any side effects. Adverse reactions are listed in both the status of the patient and in the Case Record Form (CRF). In addition, a separate registration of adverse events (AEs) for all patients in the study is filled with specific forms (Appendix 3).

Serious adverse events (SAEs) are reported within 24 hours by the investigator to the sponsor of the study, through a special form (Appendix 3).

The sponsor will determine whether there is a relation of the SAE with the study medication (side effect/ adverse reaction) and if so, whether it could be expected or not (SSAR versus SUSAR).

A SUSAR will be reported to the Ethical Board, the CBG and the competent authorities within the legally defined time frame. SSAR’s will be reported annually, in accordance with the national regulations.

Further care for patients with an adverse event will take place in line with regular patient care.

Premature withdrawal of the study: The study will be stopped prematurely if more than one SUSAR occurs in the first group of six patients, or if more than 3 SUSARs occur in the total group of 20 patients.

.

**Statistical analyses:**

Selection of the number to be included:

Initially, we choose to start the study with six patients. This limited number is motivated by the fact that the feasibility of treatment with in-hospital ACTH adminstration twice a week is not yet tested.

If the study shows good feasability, we will expand the study size to 20 patients in total. This is based on the knowlegde that in previous studies 80- 90% of patients achieved remission after treatment with ACTH.^28; 30^ However, these studies were not limited to high-risk patients. The probability that a high-risk patient achieves a spontaneous remission is around 10%.^25^ If we assume that ACTH induces remission in 60% of our high risk patients , in a randomized study with two arms, group size of 20 patients would be needed to show a difference (between spontaneous remission and ACTH-induced remission) with a power of 0.9 and an α of 0.05. While the present study contains only one arm, with 20 patients included we should be able to demonstrate a difference with a theoretical control group.

In the absence of data at final follow up (e.g. in case of early withdrawal form the study) , the latest data known as final data will be included in the analysis ( last observation carried forward).

In the analysis of the efficacy of ACTH (secondary endpoints), data from all patients enrolled will be analyzed, even if they had to discontinue treatment (intention to treat analysis). In addition a subgroup analyses of those patients who completed treatment will be performed (per protocol analysis).

**Quality control**

An independent monitor has been appointed to this study.

• Before the initiation of the study, the monitor will assess if facilities, equipment and staff are suitable to perform the study safe and properly.

• The monitor will perform a monitoring procedure every 6 months, and directly after the last patient has completed the treatment, with special attention to the informed consent forms, reporting of adverse events and the accuracy of the data on the CRF compared to the source documents.

• The monitor will report on his visits.

**Availability of source data/source documents:**

Upon request, the researchers will provide direct entry to the source data / source documents to monitors, auditors, the Ethical Board or the competent authorities.

Before study entry, patients will give written informed consent for direct access to their medical records by the above mentioned persons / entities, if requested.

**Ethical aspects**

Patients eligible for this study are those with proven kidney disease (more specific iMN) and a high risk of renal failure. Although there is no consensus in the literature about the treatment of these patients, it is generally accepted that these patients require some form of immunosuppresive treatment.

At present, the standard treatment in our center is a combination cyclophosphamide and prednisone, and treatment is started when renal function deteriorates (increase in serum creatinine). As this standard treatment is associated with many side effects, an alternative treatment is warranted.

In this current study an alternative treatment using ACTH is assessed. This agent has shown promising results in three consecutive studies of two independent groups. ^28-30^ This treatment is laready started when renal function is still normal. This means that in case of insufficient efficacy, there is still sufficient time left to treat patients with the current standard therapy. Inefficacy, defined as an increase in serum creatinine of more than 25% during treatment, is a withdrawal criterium in the study. Therefor, patients participating in the study are never at risk for delay in the start of standard treatment according to current criteria.

The administration of the study drug is by intramuscular injection by a qualified professional in a medical setting. After administration of each injection a test subject is observed for 30 minutes. The total treatment consists of 59 injections over a period of 9 months. This leads to a considerable (time) effort for the test subject. If, however, as demonstrated in previous studies, treatment with ACTH leads to a remission of the proteinuria, treatment with cyclophosphamide could be avoided.

For the group of patients this study focuses on, the effort asked seems a very reasonable price for avoiding treatment with cyclophosphamide and related side effects.

However, we admit that there is little known about the long-term outcomes after treatment with ACTH. Although we admit that there are no data on the long-term results or relapse rates after an ACTH-induced remission, even if treatment would only lead to a delay in treatment with alkylating agents, this could be an advantage for young patients who want to start a family in the near future, even if a relapse necessitates treatment with cyclophosphamide later in life.

**Data collection and handling**

Data will be captured in the following source documents:

- Medical status

- In the standardized proteinuria measurement database "Proteinuria"

From there, data will be transfered to the study Case Record Form (see Appendix 4). Some data will be directly included in this CRF, i.e. the checklist with regard to the inclusion and exclusion criteria, the registration of injections Synacthen Depot administered, and the checklist relating to the reporting of any adverse event.

The CRF is traceable to the patient by a code.

Further processing of data for analysis will take place in an Access database, and the data will be transported to an SPSS database later. The data will be processed anonymously and will not be traceable to the patient in the final study results.

**Financial aspects and insurance**

**Financial aspects:**

This is an investigator initiated study, financed by the department of Nephrology

(First line funding).

Patients participating in the study received no (financial) compensation for this.

There are no conflicting (financial) interests in this study.

**Insurance:**

In accordance with the legal regulations (WMO) an insurance has been made bij the UMC St. Radboud UMC for all subjects participating in this study. This insurance covers any damage caused by injury resulting from participation in the study, which occurs during participation in the study or within 5 years after participation. The insurance covers damage up to a maximum amount of EUR 453,780 (NLG 1 million) per subject (with the proviso that a maximum amount of Euro 6,806,703 (15 million dollars) is available to compensate all damages suffered by the study participants and a maximum amount of Euro 9,075,604 (20 million dollars) per year of insurance applies to all studies conducted by the UMC St Radboud.

The insurance does not cover the damage:

- Which occurs in offspring as a result of an adverse effect of the study on the genetic (hereditary =) material of patients;

- Of which, based of the nature of the study, it was (almost) certain that this would occur;

- Which would have also occurred if the patient would not have participated in the research.

Name and address of the insurer:

Akkermans van Elten Assurantiën BV

PO box 1275

6501 BG Nijmegen

Tel: 024 - 3511000

contact: Mr. A.J.M. Swildens

**Administration and publication policy**

The sponsor and investigator will provide an annual progress report to the Ethical Board. This will be include the report of any SSAR's. Information will be provided about the date of inclusion of the first subject, the number of patients enrolled, the number of patients who completed the study, SAE and SSAR, other problems and possible amendments.

The sponsor shall inform the competent Ethical Board within a period of 90 days after the termination of the study. The end of the study was defined as the last visit of the last patient.

If the study is terminated prematurely, the sponsor will notify the Ethical Board and the competent authority within 15 days, including notification on the reason for the termination of the study.

Results obtained from this study will be published in abstract and / or poster form or be submitted as manuscript to reputable, peer-reviewed journals. These publications will not contain any data that are directly traceable to the participating patients.

Reference List

1. Tiebosch ATMG, Wolters J, Frederik PM *et al*. Epidemiologie van primaire glomerulonefritis in de regio Zuid-Limburg. *Ned Tijdschr Geneeskd* 1986; 130: 357-360

2. du Buf-Vereijken PW, Branten AJW, Wetzels JFM. Idiopathic membranous nephropathy: outline and rationale of a treatment strategy. *Am J Kidney Dis* 2005; 46: 1012-1029

3. Ponticelli C, Zucchelli P, Passerini P *et al*. A 10-year follow-up of a randomized study with methylprednisolone and chlorambucil in membranous nephropathy. *Kidney Int* 1995; 45: 1600-1604

4. Lewis EJ. Idiopathic membranous nephropathy--to treat or not to treat? *New Engl J Med* 2008; 329: 85-89

5. Perna A, Schiepatti A, Zamora J, Giuliano GA, Braun N, Remuzzi G. Immunosuppressive treatment for idiopathic membranous nephropathy: a systematic review. *Am J Kidney Dis* 2004; 44: 401

6. Hogan SL, Muller KE, Jennette JC, Falk RJ. A review of therapeutic studies of idiopathic membranous glomerulopathy. *American Journal of Kidney Diseases* 1995; 25: 862-875

7. Cattran DC, Delmore T, Roscoe J *et al*. A randomized controlled trial of prednisone in patients with idiopathic membranous nephropathy. *New Engl J Med* 1989; 320: 210-215

8. Cameron JS, Healy MJR, Adu D. The Medical Research Council trial of short-term high-dose alternate day prednisolone in idiopathic membranous nephropathy with nephrotic syndrome in adults. The MRC Glomerulonephritis Working Party. *Q J Med* 1990; 74: 133-156

9. Branten AJW, Reichert LJM, Koene RAP, Wetzels JFM. Oral cyclophosphamide versus chlorambucil in the treatment of patients with membranous nephropathy and renal insufficiency. *Q J Med* 1998; 91: 359-366

10. Imperiale TF, Goldfarb S, Berns JS. Are cytotoxic agents beneficial in idiopathic membranous nephropathy? A meta-analysis of the controlled trials. *J Am Soc Nephrol* 1995; 5: 1543-1558

11. Cattran DC, Greenwood C, Ritchie S *et al*. A controlled trial of cyclosporine in patients with progressive membranous nephropathy. Canadian Glomerulonephritis Study Group. *Kidney International* 1995; 47: 1130-1135

12. Ponticelli C, Villa M. Does cyclosporin have a role in the treatment of membranous nephropathy? *Nephrol Dial Transpl* 1999; 14: 23-25

13. Goumenos DS, Kalliakmani P, Tsakas S, Sotsiou F, Vlachojanni JG. The remission of nephrotic syndrome with cyclosporin treatment does not attenuate the progression of idiopathic membranous nephropathy. *Clin Nephrol* 2004; 61: 17-24

14. Muirhead N. Management of idiopathic membranous nephropathy: evidence-based recommendations. *Kidney Int Suppl* 1999; 70: 55

15. Ahuja M, Goumenos DS, Shortland JR, Gerakis A, Brown CB. Does immunosuppression with prednisolone and azathioprine alter the progression of idiopathic membranous nephropathy? *American Journal of Kidney Diseases* 1999; 34: 521-529

16. Goumenos DS, Ahuja M, Davlouros P, El Nahas AM, Brown CB. Prednisolone and azathioprine in membranous nephropathy: a 10-year follow-up study. *Clin Nephrol* 2006; 65: 317-323

17. Cattran DC. Mycophenolate mofetil and cyclosporine therapy in membranous nephropathy. *Semin Nephrol* 2003; 23: 272-277

18. Branten AJW, du Buf-Vereijken PWG, Vervloet M, Wetzels JFM. Mycophenolate mofetil in idiopathic membranous nephropathy: a clinical trial with comparison to a historic control group treated with cyclophosphamide. *American Journal of Kidney Diseases* 2007; 50: 248-256

19. Ruggenenti P, Chiurchiu C, Abbate M *et al*. Rituximab for idiopathic membranous nephropathy: who can benefit? *Clin J Am Soc Nephrol* 2008; 1: 738-748

20. Fervenza FC, Cosio FG, Erickson SB *et al*. Rituximab treatment of idiopathic membranous nephropathy. *Kidney International* 2008; 73: 117-125

21. Honkanen E, Tornroth T, Gronhagen-Riska C, Sankila R. Long-term survival in idiopathic membranous glomerulonephritis: can the course be clinically predicted? *Clin Nephrol* 1994; 44: 137-139

22. Cattran DC, Pei Y, Greenwood C, Ponticelli C, Passerini P, Honkanen E. Validation of a predictive model of idiopathic membranous nephropathy: its clinical and research implications. *Kidney Ínt* 1997; 51: 901-907

23. Reichert LJM, Koene RAP, Wetzels JFM. Urinary excretion of b2-microglobulin predicts renal outcome in patients with idiopathic membranous nephropathy. *J Am Soc Nephrol* 1995; 6: 1666-1669

24. Bazzi C, Petrini C, Rizza V *et al*. Urinary excretion of IgG and a1-microglobulin predicts clinical course better than extent of proteinuria in membranous nephropathy. *Am J Kidney Dis* 2001; 38: 240-248

25. Branten AJW, du Buf-Vereijken PW, Klasen IS *et al*. Urinary excretion of b2-microglobulin and IgG predict prognosis in idiopathic membranous nephropathy: a validation study. *J Am Soc Nephrol* 2005; 16: 169-174

26. du Buf-Vereijken PW, Branten AJ, Wetzels JF. Cytotoxic therapy for membranous nephropathy and renal insufficiency: improved renal survival but high relapse rate. *Nephrol Dial Transplant* 2004; 19: 1142-148

27. Faurschou M, Sorensen IJ, Mellemkjaer L *et al*. Malignancies in Wegener's granulomatosis: incidence and relation to cyclophosphamide therapy in a cohort of 293 patients. *The Journal of Rheumatology* 2007; 35: 100-105

28. Berg AL, Nillson-Ehle P, Arnadottir M. Beneficial effects of ACTH on the serum lipoprotein profile and glomerular function in patients with membranous nephropathy. *Kidney International* 1999; 56: 1534-1543

29. Berg AL, Arnadottir M. ACTH-induced improvement in the nephrotic syndrome in patients with a variety of diagnoses. *Nephrol Dial Transpl* 2004; 19: 1305-1307

30. Ponticelli C, Passerini P, Salvadori M *et al*. A randomized pilot trial comparing methylprednisolone plus a cytotoxic agent versus synthetic adrenocorticotropic hormone in idiopathic membranous nephropathy. *American Journal of Kidney Diseases* 2006; 47: 233-240

31. Berg AL, Rafnsson AT, Johannsson M, Dallongeville J, Arnadottir M. The effects of adrenocorticotrophic hormone and an equivalent dose of cortisol on the serum concentrations of lipids, lipoproteins, and apolipoproteins. *Metabolism* 2006; 55: 1083-1087

32. Keane WF. Lipids and the kidney. *Kidney International* 1994; 46: 910-920

33. Arnadottir M. Treatment scheme ACTH. 2008.
Ref Type: Personal Communication

34. European Medicines Agency. Guidance on Good Clinical Practice (CPMP/ICH/135/95). [www.emea.eu/pdfs/human/ich/013595en.pdf](http://www.emea.eu/pdfs/human/ich/013595en.pdf) . 2006.
Ref Type: Electronic Citation

**Protocol summary**

**Titel:** Treatment with synthetic adrenocorticotropic hormone (ACTH) in patients with membranous nephropathy and high risk for renal failure. A pilot study.

**Short title:** ACTH in high risk patients with iMN.

**Eudra-CT nummer:** 2008-001647-19

**Phase:** II

**Sponsor:** University Medical Center St Radboud

**Study design:** Open, uncontrolled pilot study.

**Aim:**

Primary aim:

Evaluate the feasibility of therapy with synthetic ACTH in six patients with iMN and a high risk of kidney failure.

Secondary aims:

- To evaluate the effectiveness of ACTH in iMN, expressed as the percentage remissions achieved.
- Evaluate the effectiveness of ACTH in iMN, expressed in the change of renal function (eGFR according to MDRD) over time and the number of relapses.
- To evaluate the safety of ACTH in iMN, expressed in the number of side effects.
- To understand the mechanism of action of ACTH in iMN by assessing the effect of ACTH on endogenous cortisol / cortisone production.

**Study subjects:** Start with six patients for feasibility study.

If feasability prooved: expansion with 14 patients to 20 in total.

Inclusion criteria:

- Biopsy-proven idiopathic membranous nephropathy.
- Nephrotic syndrome: proteinuria > 3.5 g/day and serum albumine < 30 g/l
- Normal or mildly impaired renal function (eGFR > 60 ml/min, MDRD formula)
- High risk for renal failure: beta-2-microglobulin excretion > 500 ng/min
- Relative contra-indication for cyclophosphamide treatment:
  1. fertility and wish for (future) family expanding
  2. high age ( > 60 years)
  3. former cyclophosphamide treatment
  4. intolerance to cyclophosphamide

Exclusion criteria:

- Clinical,biochemical or histological signs of any underlying systemic disease
- Any infectious disease (including latent tuberculosis and/or latent amoebiasis)
- Active gastric or duodenal ulcers
- Pregnancy, lactation, inadequate contraceptives
- Clinical signs of renal vein thrombosis
- Asthma and /or any allergic conditions or hypersensitivity reactions
- Allergic reaction to synthetic ACTH in the past

**Treatment:** 1. Synacthen depot (Novartis, Basel, Switzerland) 1 mg / ml for intramuscular injection. Treatment for 9 months, according to the schedule below.

Dose increasing in a period of 8 weeksfrom 1mg once a week to 1 mg twice week.

Then treatment with 1 mg twice a week for 18 weeks. After this tapering of medication for 13 weeks.

Total number of injections per patient: 59

The injections are administered intramuscularly by a nurse or doctor. After the injection, the patient is observed for 30 minutes because of potential hypersensitivity reactions.

2. Additional supportive treatment:

• Blood pressure: target <130/80 mmHg. Preferably ACE inhibitor, if necessary. ARB

•If hypercholesterolemia exist longer than 6 months after starting treatment: add HMG-CoA reductase inhibitor. Target LDL cholesterol <2.6 mmol / l

• Moderate salt restriction

• Consider oral anticoagulation if serum albumin <20 g / l or <25 g / l with severe proteinuria (> 8 g ​​/ dg), and severe edema. Target INR 2.0- 3.0

**Wtihdrawal criteria:** 1. At the request of the patient or doctor

2. Occurence of unacceptable side effects

3. If serum creatinine increases more than 25% from baseline, or increase in serum creatinine to> 135 mg / dl. At that time, treatment with cyclophosphamide and prednisone will be started. Follow-up data on these patients will be collected according to the study protocol.

**Endpoints:**

**Primary**

• Feasibility administration of an intramuscular injection of synthetic ACTH twice a week during 9 months (% injections administered at the correct time point)

**Secondary**

- Number of patients that completes the treatment (9 months).
- Treatment burden as subjectively scored by the patients (on scale 1 to 10).
- Number of remissions, both complete and partial (proteinuria respectively. <0.2 g / dy or <2.0 g / dy)
- Number of complete remissions (proteinuria <0.2 g / dy)
- Number of partial remissions (proteinuria <2.0 g / dy)
- Change in proteinuria (if no remission achieved)
- Changes in kidney function (eGFR)
- Side effects

# Assesments: General

- Patient history and physical examination during screening for inclusion.
- Administration of study medication on schedule at the outpatient clinic or at regional hospital (dialysis unit).
- Monthly visits to doctor during the first 4 months of the study, thereafter two monthly. After the end of the treatment (9 months) visits at the end of month 12, 15, 18 and 24.

**Laboratory tests**

- Biochemical and hematological evaluation at screening and at each visit.
- Urine analysis at screening and at each visit
- “Standardized proteinuria measurement”at the start of the study and after 3 and 9 months.
- Asessement of serum and urinary cortisol at the start of the study and after 3 and 9 months

**Safety / side effects**

- After each injection, patients are observed for 30 minutes because of the possible occurrence of a hypersensitivity reaction.
- During each visit, subjects are asked about any side effects.
- Safety parameters are checked during each visit in the blood (i.e. serum potassium, liver enzymes, glucose).

**Appendix 1: PROCEDURE PROTEINURIA MEASUREMENT**

In our hospital, patients with nephrotic range proteinuria are evaluated using a standard protocol. In all these patients standardized timed urine and blood measurements are carried out as described below.

The two days prior to the measurement, the patient collects 2x 24-hour urine for the measurement of the daily excretion of creatinine, and total protein.

On the day of the measurement, patients comes to our department in fasting condition. On the morning of the measurement diuretics shoudl not be used. Patients are instructed to take 4000 mg of Sodiumbicarbonate tablets the evening before the procedure. This is to ensure that the urine pH is above 6.0, which is required for the measurement of the protein β-2-microglobulin in the urine.

The measurement is conducted by a trained research nurse. Upon arrival, patients have to drink 375 to 500 ml of tap water, to force diuresis. For the start of the measurement they are asked to urinate, after which the actual measurement starts. Patients are left for 1 hour in a horizontal position. Through an automatic blood pressure monitor blood pressure is measured, 10 consecutive measurements are made at intervals of 5 minutes and recorded (Welch Allyn, Beaverton OR, USA). After 1 hour a timed urine sample is obtained and blood withdrawal is performed. The patient is then seen by the doctor for a patient history and a brief physical examination.

From the blood sample, the following parameters are determined by standard automated techniques: sodium, potassium, calcium, phosphate, urea, creatinine, cholesterol, triglycerides, HDL-cholesterol, LDL-cholesterol (calculated), hemoglobin, hematocrit, iron, and ferritin.

The concentrations of serum albumin, transferrin and IgG are measured by using a immunonephelometrie BNII nephelometer (Behring, Marburg, Germany). Serum β-2-microglobulin is measured by ELISA [coating, code A072, detection (HRP-conjugated); code P174, both Dakopatts, Denmark].

In the urine sample total protein and creatinine are measured by standard automated techniques. The concentrations of urinary albumin, transferrin, α-1-microglobulin, and IgG are measured with immunonephelometrie and urine β-2-microglobulin is measured by ELISA.

**Appendix 2: FOLLOW-UP SCHEME**

**Visit 1**: screening for eligibility / standardized proteinuria measurement

- Patient history

- General internal physical examination

- standardized proteinuria measurement (as described in Appendix 1)

- In addition to the parameters as assesed at the standardized proteinuria measurement ASA, ALA, LDH, ACTH, cortisol and glucose are determined.

- In the 24hrs urine collected for the standardized proteinuria measurement cortisol is additionally determined

**Visit 2 at t = 0:** start study medication

- Check: informed consent signed?

- History

- Measuring blood pressure, weight, assess oedema

- Blood withdrawal ^

- Collect urine portion +

**Visit 3 at t = 1 month**

- History (with specific attention to possible side effects)

- Measuring blood pressure, weight, assess oedema

- Fasting blood withdrawal*

- collect 24hrs urine for determining creatinine, protein and cortisol

**Visit 4 at t = 2 months**

**-** History (with specific attention to possible side effects)

- Measuring blood pressure, weight, assess oedema

- Fasting blood withdrawal*

- collect 24hrs urine for determining creatinine, protein and cortisol

**Visit 5 at t = 3 months**

**-** Patient history (with specific attention to possible side effects)

- General internal physical examination

- standardized proteinuria measurement (as described in Appendix 1)

- In addition to the parameters as assesed at the standardized proteinuria measurement ASA, ALA, LDH, ACTH, cortisol and glucose are determined.

- In the 24hrs urine collected for the standardized proteinuria measurement cortisol is additionally determined

**Visit 6 at t = 5 months**

**-**  History (with specific attention to possible side effects)

- Measuring blood pressure, weight, assess oedema

- Blood withdrawal ^

- Collect urine portion +

**Visit 7 at t = 7 months**

**-**  History (with specific attention to possible side effects)

- Measuring blood pressure, weight, assess oedema

- Blood withdrawal ^

- Collect urine portion +

**Visit 8 at t = 9 months of the end of treatment**

**-** Patient history (with specific attention to possible side effects)

- General internal physical examination

- standardized proteinuria measurement (as described in Appendix 1)

- In addition to the parameters as assesed at the standardized proteinuria measurement ASA, ALA, LDH, ACTH, cortisol and glucose are determined.

- In the 24hrs urine collected for the standardized proteinuria measurement cortisol is additionally determined

**Visit 9 at t = 12 months**

**-** History (with specific attention to possible side effects)

- Measuring blood pressure, weight, assess oedema

- Blood withdrawal ^

- Collect urine portion +

**Visit 10 at t = 15 months**

**-**  History (with specific attention to possible side effects)

- Measuring blood pressure, weight, assess oedema

- Blood withdrawal ^

- Collect urine portion +

**Visit 11 at t = 18 months**

**-**  History (with specific attention to possible side effects)

- Measuring blood pressure, weight, assess oedema

- Blood withdrawal ^

- Collect urine portion +

**Visit 12 at t = 24 months: end of follow-up**

**-**  History (with specific attention to possible side effects)

- Measuring blood pressure, weight, assess oedema

- Blood withdrawal ^

**-** collect 24hrs urine for determining creatinine and protein

**Total visits: 12, including 3x 2 hours for standardized proteinurie measurement.**

**Total blood whitdrawal procedures: 12**

***** During month 1 to 12, blood is collected from a fasting patient to determine the following parameters in the blood: sodium, potassium, urea, creatinine, albumin, hemoglobin, leucocytes, thrombocytes, glucose, ASA, ALA, LDH, total cholesterol, HDL cholesterol, triglycerides, LDL cholesterol (calculated), cortisol and ACTH

**^** During t = 0 and at month 15 t / m 24, blood is collected in order to allow determining the following parameters in the blood (patient does not need to be fasted): sodium, potassium, urea, creatinine, albumin, hemoglobin, glucose , total cholesterol

**+** In the urine portion protein / creatinine ratio is determined

**Appendix 3: REPORT AND EVALUATION OF ADVERSE EVENTS (AE) AND SERIOUS ADVERSE EVENT (SAE)**

During each visit, subjects are asked about any side effects. Adverse reactions are listed in both the clinical file of the patient and in the Case Record Form (CRF).

For classification of adverse effects, the definitions according to the GCP guideline are used

Additional registration ( next to status and CRF) of adverse events (AEs) for all patients in the study is performed using the forms below.

Serious adverse events (serious adverse events, SAEs) are reported within 24 hours by the investigator to the sponsor.

The sponsor determines whether there is a relation of the SAE with the study medication and if so, whether there is an expected or unexpected adverse reaction (SSAR versus SUSAR).

A SUSAR will be reported to the Ethical Board, the CBG (cq. Eudra Vigilance) and the competent authority (CCMO) within the legally defined time frame of 15 days (7 days in case of a life-threatening or fatal events).

SSAR’s will be reported annually to the Ethical Board and the CCMO.

Further care for patients with an adverse event will take place in line with regular patient care..

The actions taken in response to a (S)AE will be listed in the patient’s clinical file and the CRF.

**AE Registration Form for protocol** ***“*Treatment with synthetic adrenocorticotropic hormone(ACTH) in patients with membranous nephropathy and high risk for renal failure. A pilot study.”**

| Subjectnr:  Event:  Event nr.: | ________  AE  ________ | Initials: __________  Initial report/ Follow-up report |
| --- | --- | --- |

**AE_Name** Name of Adverse Event _______________________

________________________

________________________

**AE_Number** Number of AE ___

**AE_Start** Start date and time of AE __________________

**AE_Stop** Stop date and time of AE ___________________

**AE_Ongoing** Ongoing Adverse Event 0 (No)

1 (Yes)

! (Missing)

**AE_Sympt** Symptoms of Adverse Event ________________________

________________________

________________________

________________________

**AE_Action** Action taken on AE 1 (None)

2 (Dose adjustment)

3 (Temporary stop)

4 (Permanent stop)

! (Missing)

| Subjectnr: | **Initials:** | **Event:** AE | **Event nr.** |
| --- | --- | --- | --- |

**AE_Action** Action taken on Adverse Event 1 (none)

2 (dose adjustment)

3 (temporary stop)

4 (permanent stop)

! (Missing)

**AEAddTreat** Additional treatment of AE 1 (no)

2 (medication: complete CRF)

3 (other: describe in note)

! (Missing)

**AE_AddTreat1** If other treatment, specify _________________________________________________________

_________________________________________________________

_________________________________________________________

**AERelation** Relation of AE to study medication? 1 (not related)

2 (unlikely related)

3 (possibly related)

4 (probably related)

5 (definitely related)

! (Missing)

**AE_Intensity** Intensity of AE according to NCI CTC _____

**AE_Severity** Severity of Adverse Event 1 (mild)

2 (moderate)

3 (severe)

**AE-SAE** Is this a Serious AE 0 No

1 Yes

If Yes, reason:

1 (Prolonged) hospitalization

2 Life-threatening

3 Persistent or significant disability or incapicity

4 Congenital anomaly or birth defect

5 Death

**If SAE complete SAE form and report!**

Reported on: _______________________ (date)

By: ________________________ (name investigator)

_________________________ (signature)

**SAE Registration Form for protocol** ***“*Treatment with synthetic adrenocorticotropic hormone(ACTH) in patients with membranous nephropathy and high risk for renal failure. A pilot study.”**

| Subjectnr:  Event:  Event nr.: | ________  SAE  ________ | Initials: __________  Initial report/ Follow-up report |
| --- | --- | --- |

**SAE** Has any SAE taken place? 0 (No)

1 (Yes)

! (Missing)

**SAE_Name** Name of Serious Adverse Event _______________________

________________________

________________________

**SAE_Number** Number of SAE ___

**AE_Number** Number of Adverse Event ___

**SAE_Action** Action taken on SAE 1 (None)

2 (Dose adjustment)

3 (Temporary stop)

4 (Permanent stop)

! (Missing)

**SAE_AddTreat** Additional treatment on SAE 1 (No)

2 (Med: compl ConMedForm)

3 (Other: describe in note)

! (Missing)

**SAE_AddTreat1** If other treatment, specify _________________________________________________________

_________________________________________________________

_________________________________________________________

_________________________________________________________

**SAE_Relation** Relation of SAE to studymedication? 1 (Not likely)

2 (Unlikely related)

3 (Possibly related)

4 (Probably related)

5 (Definitely related)

! (Missing)

| **Subjectnr:** | **Initials:** | **Event:** SAE | **Event nr.** |
| --- | --- | --- | --- |

**SAE_Start** Start date and time of SAE __________________

**SAE_Stop** Stop date and time of SAE ___________________

**SAE_Ongoing** Ongoing Serious Adverse Event 0 (No)

1 (Yes)

! (Missing)

**SAE_ActStart** Start action taken on SAE (date) __________________

**SAE_ActStop** Stop action taken on SAE ___________________

**SAE_Expected** Could the SAE be expected? If SAE 0 (No)

might be related and is unexpected, 1 (Yes)

complete SUSAR form and report ! (Missing)

immediately according to SOP.

­­­­­­­­­­­­­

Reported on: _______________________ (date)

By: ________________________ (name investigator)

_________________________ (signature)

Received and processed on: _______________________ (date)

By: ________________________ (name sponsor)

_________________________ (signature)

**In case of SUSAR:**

Reported to: METC ____________ (date)

CCMO ____________ (date)

CBG _____________ (date)

EudraVigilance _____________ (date)

# 
