# Supplementary material for: Synthetic ACTH in High Risk Patients with Idiopathic Membranous Nephropathy: A Prospective, Open Label Cohort Study
Source: PLoS One. 2015 Nov 12;10(11):e0142033. doi: 10.1371/journal.pone.0142033 (PMC4642982; doi:10.1371/journal.pone.0142033)
Supplement: S1 Table — (DOCX) [file pone.0142033.s003.docx]

**S1 Table: Dose and amount of synthetic ACTH injections**

| **Week 1-4** | **Week 5-8** | **Week 9-26** | **Week 27-30** | **Week 31-34** | **Week 35-39** |
| --- | --- | --- | --- | --- | --- |

1/wk 1 mg **n=4**

**n-**

3/2 wk 1 mg **n=6**

n

2/wk 1 mg **n=36**

3/2wk 1mg **n=6**

1/wk 1 mg **n=4**

1/2wk 1mg **n=3**

**Dose and amount of injections (n)**
