# Supplementary material for: Synthetic ACTH in High Risk Patients with Idiopathic Membranous Nephropathy: A Prospective, Open Label Cohort Study
Source: PLoS One. 2015 Nov 12;10(11):e0142033. doi: 10.1371/journal.pone.0142033 (PMC4642982; doi:10.1371/journal.pone.0142033)
Supplement: S2 Table — (DOCX) [file pone.0142033.s004.docx]

**S2 Table: Outcomes ACTH treated patients vs. all cyclophosphamide treated patients**

|  | **ACTH group** | **CP group n=53** | **P value** |
| --- | --- | --- | --- |
| **Remission after first therapy** | 11/20 (55 %) | 49/53 (92 %)* | 0.001 |
| **Additional treatment at end of follow-up** | 10/20 (50 %) | 11/52 (21 %)** | 0.021 |

^CP= cyclophosphamide^ **^*^**^Between 1998 and 2007 100 patients were treated with our standard protocol of CP and steroids. Fifty-three had a creatinine level <135 µmol/l at the start of immunosuppressive treatment, which was one of the inclusion criteria in the ACTH group. **From one patient this information is not available.^
